# Fixture design effects on posterior dental implant stability using finite element analysis (FEA): a systematic review

**DOI:** 10.1186/s13005-025-00566-x

**Published:** 2025-11-24

**Authors:** Kriswanto Kriswanto, Jamari Jamari, Rachael Andika, Athanasius Priharyoto Bayuseno, Abdulfatah Abdu Yusuf, Muhammad Imam Ammarullah

**Affiliations:** 1 Department of Mechanical Engineering, Faculty of Engineering, Universitas Diponegoro, Semarang, Central Java 50275 Indonesia; 2 Department of Mechanical Engineering, Faculty of Engineering, Universitas Negeri Semarang, Semarang, Central Java 50229 Indonesia; 3 Undip Biomechanics Engineering & Research Centre (UBM-ERC), Universitas Diponegoro, Semarang, Central Java 50275 Indonesia; 4 Department of Mechanical Engineering, College of Engineering, University of Liberia, Monrovia, Montserrado 1000 Liberia; 5 Bioengineering and Environmental Sustainability Research Centre, University of Liberia, Monrovia, Montserrado 1000 Liberia

**Keywords:** Fixture design, Posterior, Dental implant, Stability, Finite element analysis (FEA), Patient

## Abstract

Tooth loss is prevalent in the posterior region, and dental implants require optimized fixture design to ensure biomechanical stability, which is often the approach using finite element analysis (FEA). This systematic review, using Preferred Reporting Items for Systematic Reviews and Meta-Analyses (PRISMA) 2020 guidelines in its writing, aimed to assess the influence of fixture design on posterior implant biomechanical stability, such as stress and strain, by FEA analysis. The study search used PubMed, ScienceDirect, MDPI, SpringerLink, and OpenAire databases to find studies that analyzed fixture level, body shape, thread characteristics, diameter, length, and neck model, covering studies published between 2020 and 12 January 2025. Data from 39 of the 3,562 studies were synthesized narratively, guided by SWiM, and grouped by design parameters, with bias assessed using ROBFEAD. Tissue-level fixtures outperformed bone-level fixtures in reducing cortical bone stress and improving fatigue resistance, while the tapered body shape increased stress distribution in low-density bone (D4). Square threads minimize micro stress and movement, and larger diameters and lengths increase stability, although excessive dimensions increase cortical stress. The micro-thread neck design reduces bone resorption. The high risk of bias resulting from the lack of mechanical validation, isotropic assumptions, and static loading conditions requires attention when interpreting research findings. Clinical or experimental validation is required. Future studies should standardize thread reporting, use computed tomography (CT)-based modeling, and simulate dynamic loading for patient-specific designs.

## Introduction

Tooth loss substantially impacts a person’s quality of life, especially interfering with important oral functions such as mastication and speech [1–4]. Dental caries, periodontal disease, malocclusion, systemic disorders, trauma, tooth impaction, smoking, drug use, and aging can be factors that contribute to tooth loss [5–10]. Dental caries are caused by the prolonged interaction between acidogenic bacteria, fermentable carbohydrates, and saliva, which damage tooth enamel [11]. Periodontal disease, a chronic inflammatory condition affecting the supporting structures of the teeth, arises from the body’s immune response to subgingival biofilms [12]. Malocclusion, a misalignment between the upper and lower dental arches, is caused by many factors, such as injuries, health conditions, habits, and economic level [13, 14]. Furthermore, a higher incidence of edentulism has been linked to systemic conditions such as diabetes mellitus, hypertension, cardiovascular disease, and asthma [15].

Dental trauma, such as accidents, sports injuries, and falls, can also cause tooth loss, with the severity depending on the direction and magnitude of the force, object geometry, and tissue resilience [16]. Impaction is when teeth fail to erupt into the dental arch in time due to limited eruption space or early loss of primary teeth [17, 18]. Behavioral factors such as smoking and illegal drug consumption further exacerbate the risk of possible tooth loss [10]. At Riyadh Elm University Hospital, individuals aged 35–50 years showed the highest incidence of edentulism [6]. Epidemiological data from dental clinics in the Faculty of Dentistry, Sana’a University, and Al-Murtada private clinics shows that posterior teeth are more commonly affected by tooth loss than anterior teeth, with molars accounting for 69.85% and premolars accounting for 21.07% of cases. In contrast, incisors and canines accounted for only 6% and 3.08% respectively [7]. At Riyadh Elm University Hospital, caries were the dominant factor in cases of molar (65.95%), premolar (53.75%), and anterior (37.9%) tooth loss. In comparison, pre-prosthetic treatment was more dominant in the anterior area (22.1%) than the premolar (14.90%) and molar (5.95%). In contrast, periodontal disease is more dominant in the anterior area (31.85%) compared to molars (14.75%) and premolars (20.10%) [6]. Tooth loss will coincide with dental implants, with the posterior mandibular region experiencing the highest frequency, followed by the posterior maxillary region, anterior maxillary region, and anterior mandibular region [19].

Edentulism can be treated with either space closure or space reopening strategies. Space reopening includes autotransplantation, prosthetic rehabilitation, and implant placement. Meanwhile, space closure is usually achieved by placing orthodontic fixtures [20]. Dental implants are widely preferred among these modalities due to superior aesthetics, functional restoration, and biomechanical reliability. The advantage of dental implants is that they can osseointegrate with the peri-implant bone, where osseointegration is a direct structural and functional connection between the bone and the implant (fixture) surface essential for load-bearing and long-term survival. In addition, the implant does not interfere with the bone remodeling process, and if osseointegration fails, it can be removed without significant damage to the bone [21]. Therefore, dental implants show a 10-year survival rate of 99.4% in healthy individuals and 95.9% in patients with systemic conditions [22].

Implant stability is influenced by various factors, including the quality of osseointegration, implant material, bone quality, type of abutment-implant connection, and fixture design [23–25]. Biocompatible materials are essential to minimize immunogenic responses and ensure long-term integration [26]. These materials are broadly classified as tolerant, which induces the process of osteogenesis by the formation of fibrous tissue between the implant and bone, which provides direct bonding between bone and implant without any chemical reaction between the material and surrounding tissues and fluids, and bioactive which provides direct chemical bonding between implant and bone by direct deposition of bone matrix on the implant material [27]. Titanium and its alloys are widely used due to their good mechanical properties and biocompatibility, resulting in survival rates between 97% and 99% that can last more than 10 years [28]. Bone density is another determinant of the bone quality aspect of implant primary stability, and there are many classifications, including cortical-trabecular composition and Hounsfield Unit (HU) value [29, 30]. Based on bone composition, there are four types of bone, ranging from homogeneous cortical (Type 1) to thin cortical bone with low-density trabecular (Type 4) [31]. While HU-based criteria identify five categories ranging from D1 (HU >1250) to D5 (HU < 150) [32]. In addition, the abutment-implant connection is important in minimizing macroscopic gaps and bacterial infiltration to implant failure, with conical connections offering superior sealing and biomechanical stability compared to external or internal connections [33, 34]. Fixture design is one of the most critical elements in ensuring implant stability and long-term function, where the fixture as a component embedded in the alveolar bone acts as a substitute for the natural tooth root and plays a role in stress transfer at the bone-implant interface and osseointegration [35, 36]. Fixture design parameters such as shape, diameter, length, and thread geometry (depth, pitch, width, angle) play an important role in determining the success and stability of dental implants for each case of edentulism [24].

Finite element analysis (FEA) has emerged as a non-invasive numerical approach widely used to assess biomechanical reactions in dental implant studies. Discretizing complex anatomical models into smaller elements connected by nodes and composed of mathematical equations allows predictive modeling of biomechanical responses under various boundary conditions [37]. FEA offers several advantages, including cost-effectiveness, time efficiency, and the ability to visualize mechanical performance in detail [38]. However, FEA cannot fully replace experiments or clinical trials due to its inability to replicate biological complexities, such as oral pH fluctuations, temperature changes, biofilm formation, and immune responses, which require simplification [39, 40]. Therefore, FEA findings should be validated with clinical or experimental data to ensure accuracy in patient care applications in various conditions [41].

Previous systematic reviews have explored the influence of implant diameter and length on biomechanical outcomes, with the consensus indicating a more substantial influence of diameter on implant stability [42]. Other reviews have attempted to examine long-term biomechanical performance using FEA with dynamic loading. However, no agreement was reached on the optimal fixture design due to the many influencing variables [43]. These reviews have limitations in that they incorporate heterogeneous implant locations (anterior and posterior) without subgroup analysis and do not comprehensively evaluate key fixture design parameters. No systematic review has explicitly focused on evaluating the biomechanical impact of detailed fixture design parameters such as fixture level, fixture body shape, thread shape, thread depth, thread width, thread pitch, thread angle, fixture diameter, fixture length, and neck model on posterior dental implants using FEA. This region is crucial as it endures the highest masticatory forces and is the most common site for implant placement. This knowledge gap requires a focused review to integrate current findings and guide evidence-based implant design. Therefore, this systematic review aims to assess the influence of fixture design on stress and strain distribution in posterior dental implants using FEA. The findings are expected to assist clinicians and researchers in optimizing implant selection, thereby improving long-term outcomes in posterior restorations.

## Materials and methods

This systematic review was conducted using the Preferred Reporting Items for Systematic Reviews and Meta-Analyses (PRISMA) 2020 statement [44] and the Synthesis Without Meta-analysis (SWiM) reporting guideline [45]. The research question was framed using population, intervention, comparison, and outcome (PICO) [46]: “How does fixture design in single dental implants placed in posterior edentulous regions influence biomechanical stability, as determined by FEA?” Details appear in Table 1.

**Table 1:** PICO and the details

PICO Element	Description
Population (P)	Patients with posterior edentulism
Intervention (I)	Single dental implant
Comparison (C)	Fixture level, fixture body shape, thread shape, thread depth, thread width, thread pitch, thread angle, fixture diameter, fixture length, and neck model
Outcome (O)	Biomechanical stability metrics (such as von Mises stress) via FEA

### Inclusion and exclusion criteria

The inclusion criteria for this study were: (1) articles published in English; (2) studies employing FEA as the primary method of biomechanical evaluation; (3) studies explicitly focusing on the posterior dental region (molars or premolars); and (4) studies investigating fixture designs in single dental implants.

The exclusion criteria were: (1) articles published in languages other than English; (2) literature reviews of any kind, including systematic, narrative, or other types of literature reviews; (3) studies that did not specify the anatomical location of implant placement; and (4) studies evaluating devices other than single dental implants, such as orthodontic mini-implants, overdentures, bridges, All-on-X systems, or clear aligners. To ensure consistency of study design and comparability of outcomes and to isolate the relationship between fixture-design parameters and biomechanical responses, we limited inclusion to in-silico finite element analyses (FEA). Clinical and experimental studies were excluded because their heterogeneous protocols (such as surgical techniques, prosthetic designs, loading regimens, follow-up durations, and patient factors) preclude valid like-for-like synthesis with FEA. This decision was prespecified and reported following PRISMA 2020 and SWiM guidance for transparent synthesis within a single design class [44, 45].

### Search strategy

The search was conducted systematically across four primary electronic databases, PubMed, ScienceDirect, MDPI, and SpringerLink, covering publications from 2020 to 12 January 2025. In addition, grey literature was retrieved via OpenAIRE to broaden the range of information sources. Primary search terms for primary electronic databases and grey literature included “dental implant,” “finite element analysis,” “posterior,” “stability,” “design,” “dental implant length,” “dental implant diameter,” “dental implant thread design,” and “dental implant neck wall thickness,” together with relevant synonyms and variations. The strategy was tailored to each database’s specifications using Boolean operators AND and OR. For the content type, the filter displays SpringerLink only scientific articles.

### Study selection

The records found using the search strategy were imported into EndNote 21 (web.endnote.com) On 16 January 2025 and deduplicated using the software’s default exact-matching settings. The resulting dataset was exported to Microsoft Office Excel Professional Plus 2019 on 18 January 2025 for further processing, which included manual deduplication in Excel by applying exact matching to the title, first author, and publication year fields. Before the full screening, a pilot review of titles and abstracts using a 10% sample was conducted to assess inter-reviewer agreement. Thereafter, the reviewers screened titles and abstracts independently, and full texts of studies passing the initial screening were assessed. A record of the number of included and excluded articles and detailed reasons for full-text exclusion were reported. Resolution of disagreements will be by discussion until consensus is reached and, if necessary, by review from a third reviewer.

### Extraction data and study characteristics

Upon completion of the study selection, the reviewers extracted data independently using a predesigned extraction form aligned with the review objectives. The extraction encompassed six main categories:

1. Bone characteristics, including fixture placement site, bone classification, bone layers, and computed tomography (CT) imaging.
2. Fixture characteristics include covering fixture material, commercial product–based model, fixture brand, abutment–fixture connection, fixture level, fixture body shape, and fixture thread shape.
3. Fixture dimensional characteristics comprise fixture diameter, fixture length, thread width, thread depth, and thread pitch.
4. Loading characteristics, namely load type, axial load, lateral load, oblique load, area of load application, and additional loading.
5. FEA characteristics, consisting of FEA type, element shape, convergence testing, and model characteristics; and.
6. Study details and findings encompass additional methods, simulation components, independent variables, dependent variables, boundary conditions, and main findings.

When a study did not explicitly report a datum but it could be inferred from context, the item was marked with an asterisk (*); if data were absent, the field was labeled “ND” (Not Described). All extracted results were tabulated according to the predefined categories. Discrepancies in extraction were resolved through discussion until a consensus was reached. After extracting the data, we organized the study characteristics for analysis. We used Tableau Public Edition (version 2025.1.0) for data visualization and further analysis. The visual outputs included bar charts, pie charts, and tables. We calculated the percentage distribution of relevant variables to derive meaningful insights from the data.

### Quality assessment

Risk of bias in studies using finite element analysis in dentistry (ROBFEAD) is an assessment instrument used to perform quality assessment. It is an instrument developed and validated to assess the quality of in silico FEA studies in dentistry [47]. ROBFEAD comprises 22 structured questions grouped into six principal domains: development of model, material properties, impact load, endpoints tested, mechanical validation, and reporting error (Table 2). Each item was scored as “Yes,” “No,” or “Not Applicable.” Negative responses (“No”) are weighted according to their significance, and studies are then classified as low, moderate, or high risk of bias by the following criteria:

- Low risk of bias: if it contains only one “No” response in a question within a low-risk domain;
- Moderate risk of bias: if it contains at least one “No” in a moderate-risk domain or two or more “No” responses in low-risk domains.
- High risk of bias: if it contains one or more “No” responses in a high-risk domain, two or more “No” responses in moderate-risk domains, or a combination of two “No” responses in low-risk domains and one “No” in a moderate-risk domain.

In line with the objective of this systematic review, which examined the influence of implant fixture design on stress and strain distribution in the posterior region, minor modifications were made to the original ROBFEAD tool. Anatomical sub-structures irrelevant to the study scope (such as enamel, pulp, and soft tissue) were excluded, and specific guiding questions were revised to enhance clarity and contextual relevance. These refinements ensured consistency with PRISMA 2020 and SWiM reporting standards.

Validation of the modifications was performed in two steps. First, two reviewers (with expertise in biomechanics and FEA) independently applied the adjusted questions to a pilot set of five studies. Second, any discrepancies were discussed until full consensus was achieved. These processes confirmed that the modification could improve clarity without introducing bias. No calibration sessions or statistical inter-reviewer reliability tests were performed, but reviewers strictly adhered to the ROBFEAD criteria.

**Table 2:** Guiding questions and corresponding risk categories in the ROBFEAD instrument

Domain	No	Guiding Question	Risk Category
Model development	1	Was a 3D model developed using DICOM images?	High
	2	Were all the sub-structures as relevant to the study defined? (cortical and cancellous bone) AND/OR adequate justification has been given for the anatomical structures not defined in the area of interest.	High
	3	Were realistic dimensions of sub-structures described?	Moderate
	4	Were appropriate boundary conditions/restraints of the structure and sub-structures adequately explained?	Moderate
	5	Was convergence testing done during generation of model?	Moderate
	6	Were appropriate contact condition between interfaces of the sub-structures defined? (friction/frictionless/bonded)	High
Material properties	7	Were appropriate properties given to different sub-structures? (Cortical bone and cancellous bone- anisotropic, transversely isotropic, orthotropic)	High
	8	Was appropriate elastic behavior of each sub-structures defined? (linearly elastic/non-linearly elastic)	Moderate
	9	Were the values of Poisson’s ratio and Young’s modulus of each sub-structure mentioned with reference or calculated using radio-density?	High
	10	Were age-appropriate properties described for the biological sub-structures as per the clinical context?	Low
Impact load	11	Were dynamic loading conditions applied? (if applicable)	High
	12	Was the range of force appropriate for the study purpose?	High
	13	Was the point/s of application of force appropriate for the study purpose?	Moderate
Endpoints tested	14	Is the endpoint tested appropriate for the study purpose? (von Mises stress/Max Principle stress/Max Shear stress/Displacement, and others)	Moderate
Mechanical validation	15	Was the validation of test results done using appropriate mechanical model?	Low
Reporting error	16	Are points such as shape of elements, number of elements and nodes described?	Low
	17	Is appropriate detailing of different types of models or variable used in the study mentioned?	Low
	18	Are the software used for the model synthesis and mesh development mentioned with details of the version?	Low
	19	Are the software used for the Finite element analysis mentioned with details of the version?	Low
	20	Are study results described as per the objectives?	Moderate
	21	Is clinical relevance of the results described?	Moderate
	22	Is limitation of the FEA model/study described?	Moderate

### Data synthesis

Data from the selected studies were qualitatively synthesized and organized in thematic tables. Meta-analysis could not be performed because the studies did not report statistical parameters important for meta-analysis, such as standard deviation or sample size. Therefore, a narrative synthesis was performed by grouping the studies based on implant fixture design characteristics, such as fixture level, fixture body shape, thread shape, thread depth, thread width, thread pitch, thread angle, fixture diameter, fixture length, and neck model.

The synthesis followed the PRISMA 2020 and SWiM guidelines, explaining the clustering methods and criteria. Data visualization techniques were employed to enhance the clarity and interpretation of the findings. Data visualization techniques were used to improve the findings’ readability and clarity. To better grasp the outcomes, this SWiM approach combines structured clustering, narrative synthesis, and visual depiction. It offers a broad overview of variations in fixture design and their biomechanical implications for stress distribution and the stability of dental implants.

## Results

### Study search and study selection

The study search was performed in five databases, PubMed, ScienceDirect, MDPI, SpringerLink, and OpenAIRE, to uncover papers pertinent to the impact of dental implant fixture design on stress and strain distribution in the posterior area. The search produced 3,562 articles, with contributions from SpringerLink (26.8%), PubMed (22.2%), OpenAIRE (18.4%), ScienceDirect (16.3%), and MDPI (16.2%). The search strategies, which combine Boolean operators and specific keywords with customization to each database, are detailed in Table 3.

**Table 3:** Boolean operators and study search results

Electronics database	Boolean Operator	Study identified
PubMed	((((((((((((((((dental stress analysis[MeSH Terms]) AND (dental implants*[MeSH Terms])) AND (finite element analysis[MeSH Terms])) AND (finite element analysis) AND (design)) AND (stability)) AND (posterior)) OR (molar)) OR (premolar)) AND (dental implant diameter)) OR dental implant length)) OR (dental implant thread design)) NOT (systematic review)) NOT (retrospective)) NOT (meta-analysis)) NOT (randomized controlled trial)) NOT (review)	792
ScienceDirect	Title, abstract, keywords: (dental implant) AND (finite element analysis) AND (design) AND (stability) AND (posterior) OR (dental implant diameter) OR (dental implant length) OR (dental implant thread design) OR (dental implant neck wall thickness);Title: NOT (systematic review) NOT (retrospective) NOT (meta-analysis) NOT (randomized controlled trial) NOT (review)	215
MDPI	All Fields: (dental implant*) AND (finite element analysis) AND (stability) AND (posterior) AND (design) OR (dental implant length) OR (dental implant diameter) OR (dental implant thread design) OR (dental implant neck wall thickness)	231
SpringerLink	(dental implant) AND (finite element analysis) AND (design) AND (stability) AND ((posterior) OR (molar) OR (premolar)) OR (dental implant diameter) OR (dental implant length) OR (dental implant thread design) OR (dental implant neck wall thickness) NOT (systematic review) NOT (retrospective) NOT (meta-analysis) NOT (randomized controlled trial) NOT (review)	956
OpenAIRE	Any Field: Includes: (dental implant) AND (finite element analysis) AND (stability) AND (posterior) AND (design) OR (dental implant length) OR (dental implant diameter) OR (dental implant thread design) OR (dental implant neck wall thickness)	1368
Total study		3562

**Fig. 1:**
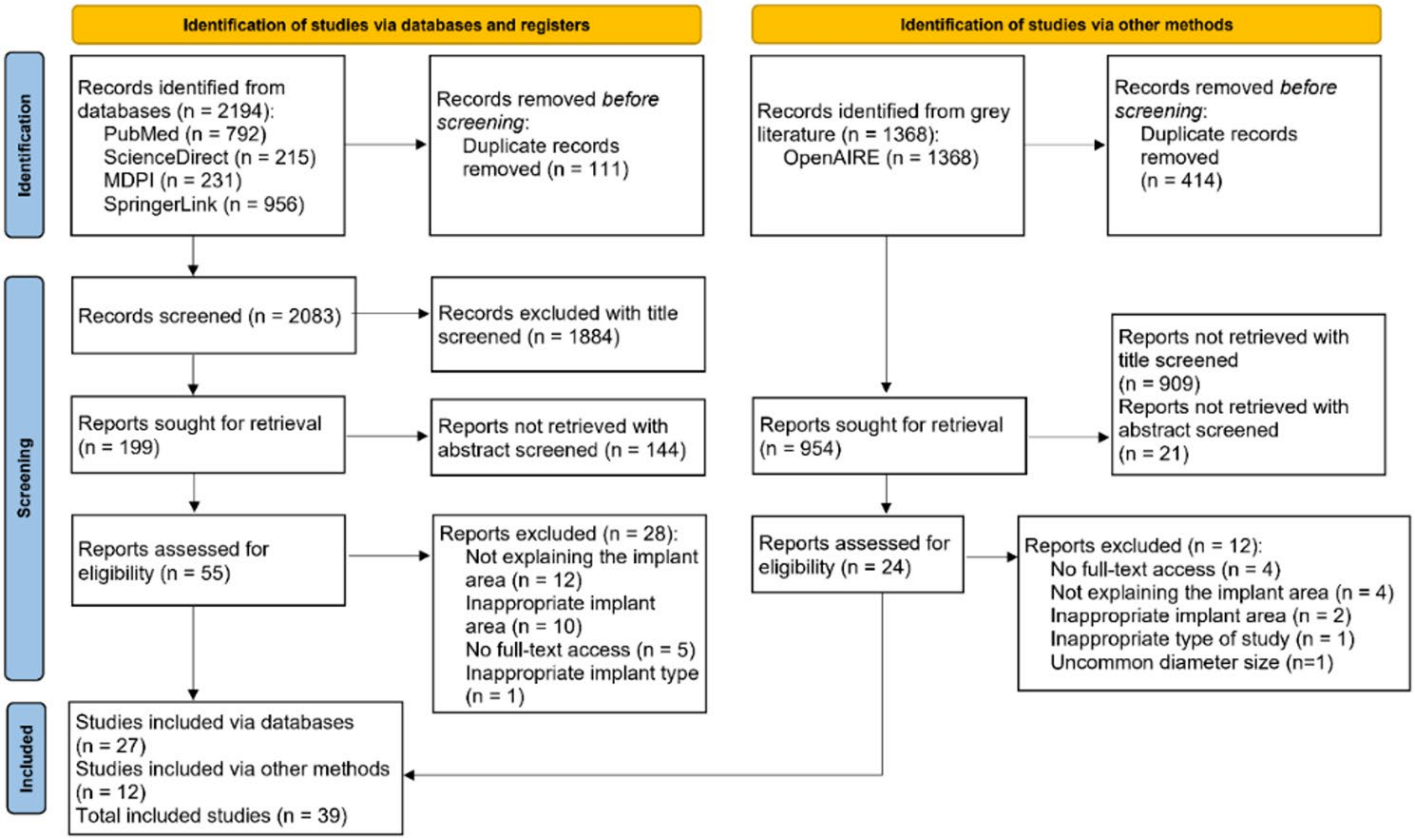
PRISMA 2020 flow diagram study selection

Following deduplication, 525 duplicate articles were removed, leaving 3,037 unique records for screening. The selection process adhered to PRISMA 2020 guidelines, with title screening excluding 2,793 records and abstract screening excluding 165 records that did not meet inclusion criteria, such as irrelevant to study about dental implant fixture design or FEA. A full-text assessment of the remaining 79 articles led to the exclusion of 40 articles, primarily not explaining or inappropriate placement sites of fixtures and inaccessible full texts. Restricting inclusion to English-language articles with accessible full texts may have constrained the representation of study on fixture design for posterior stress distribution, potentially introducing selection bias. Ultimately, 39 articles were included for data extraction and synthesis, as illustrated in the PRISMA flowchart (Fig. 1).

### Extraction data and study characteristics

**Fig. 2:**
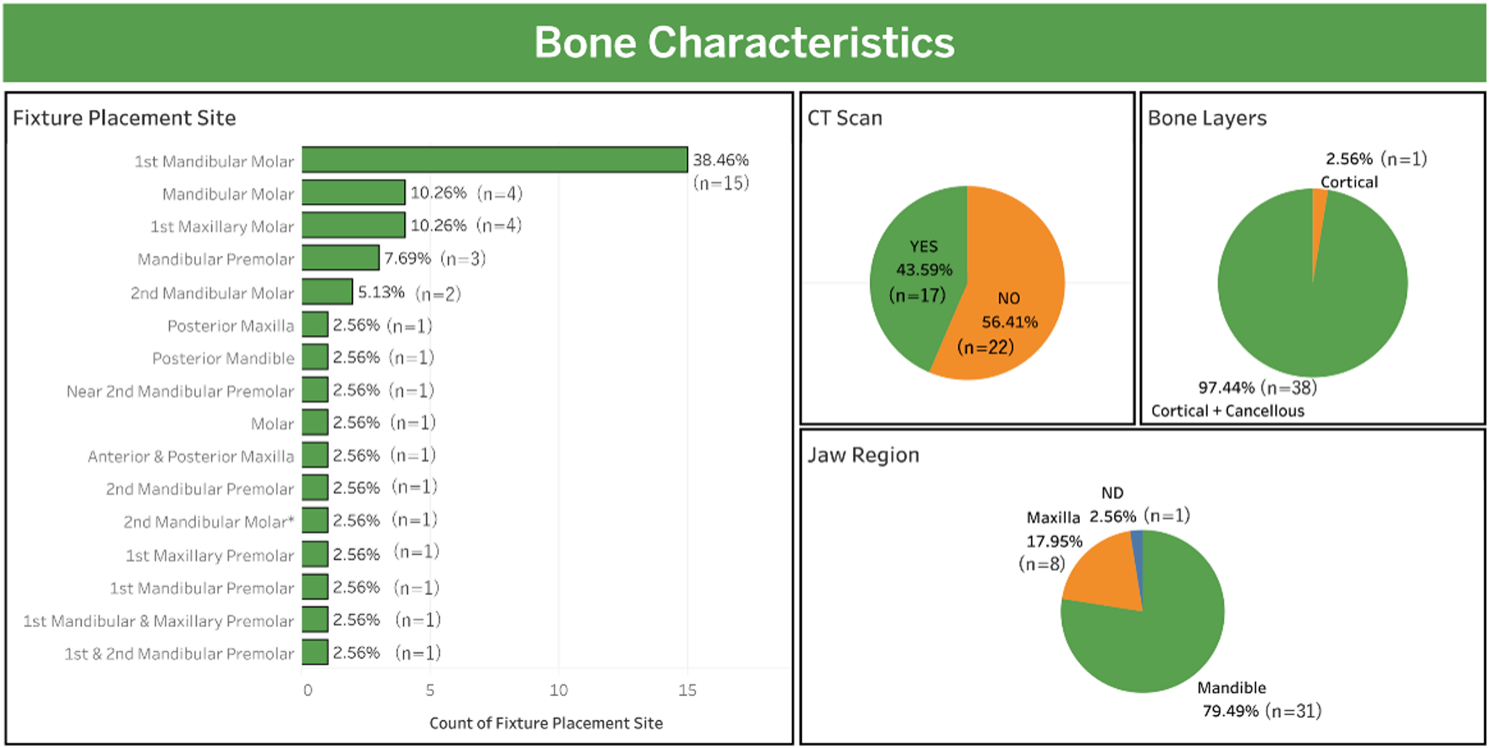
Bone characteristics of the study

**Fig. 3:**
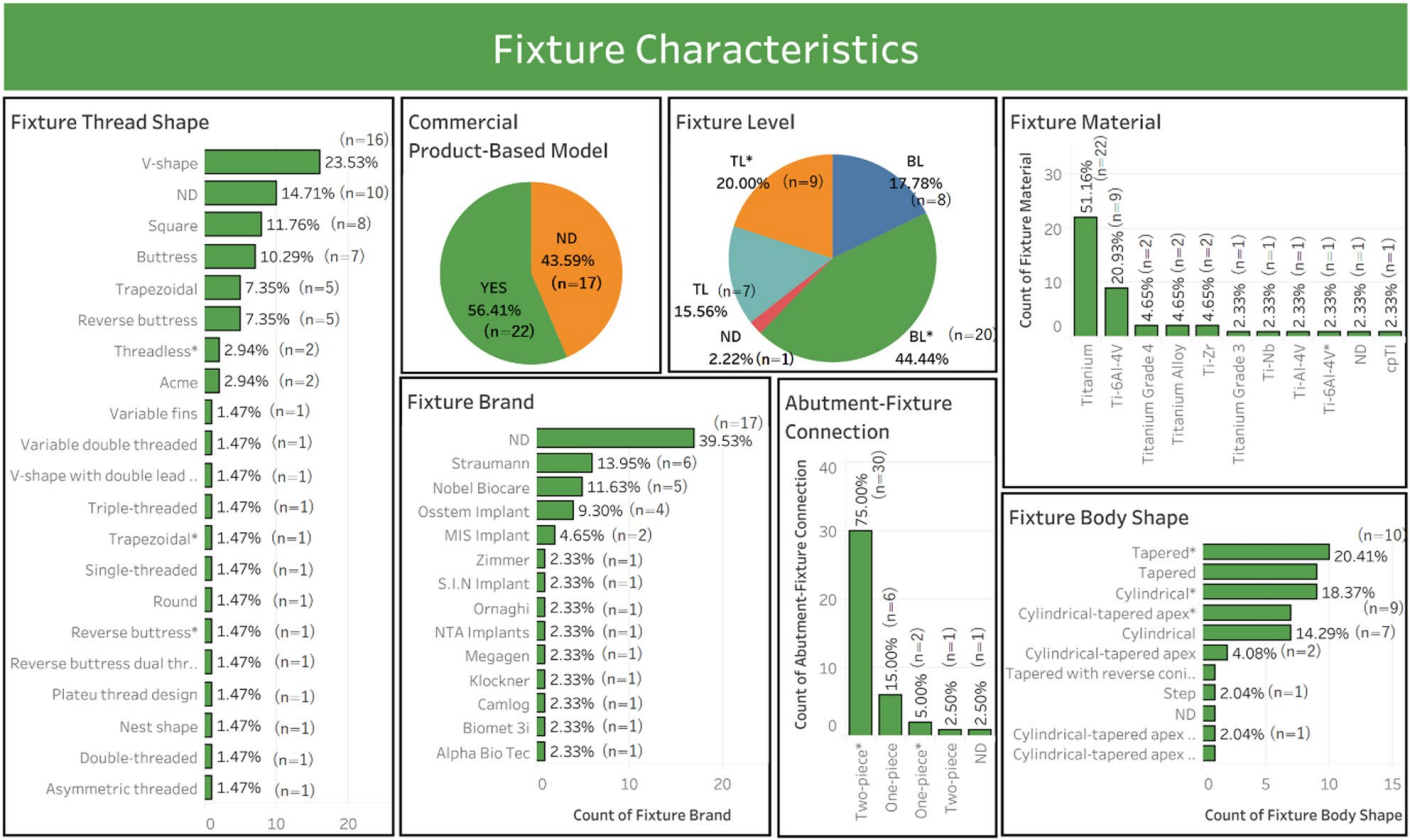
Fixture characteristics of the study

**Fig. 4:**
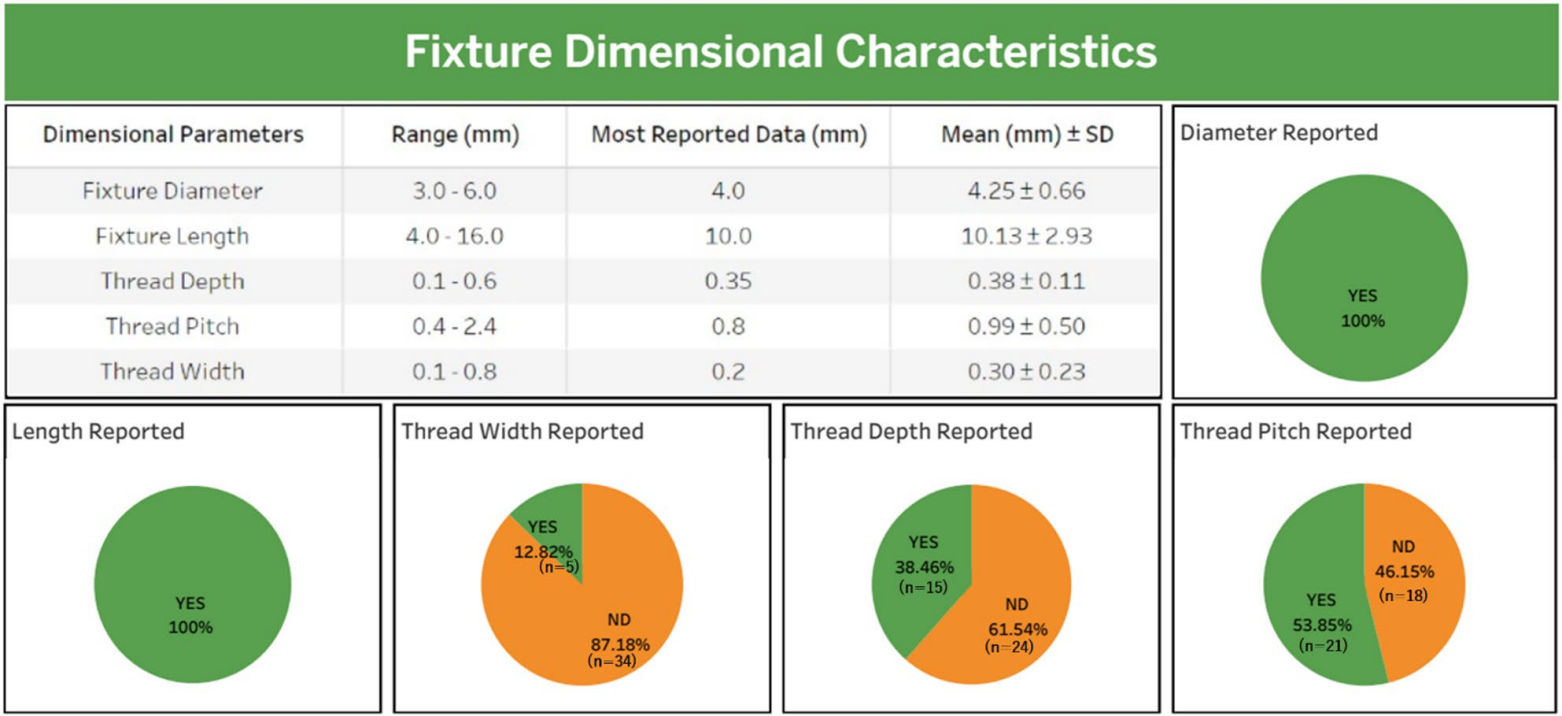
Fixture dimensional characteristics of the study

**Fig. 5:**
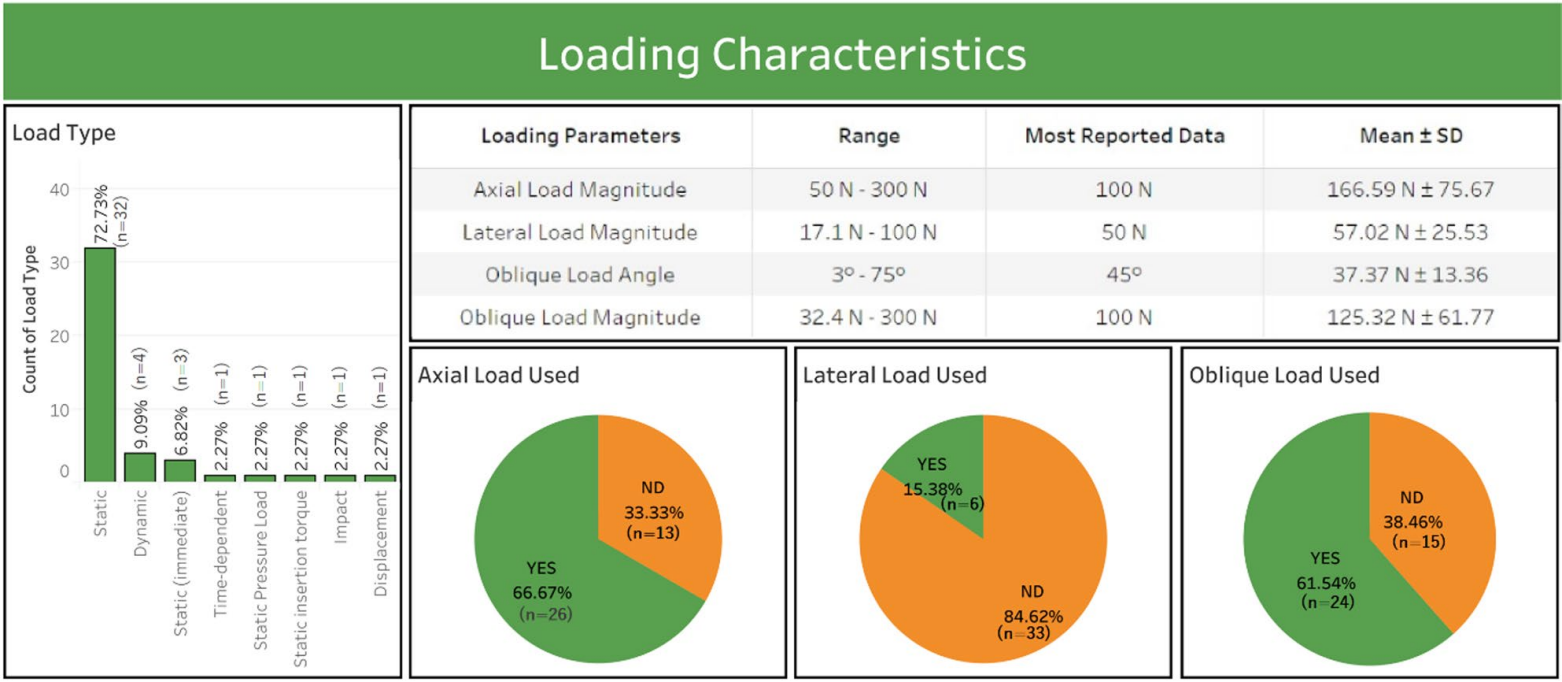
Loading characteristics of the study

**Fig. 6:**
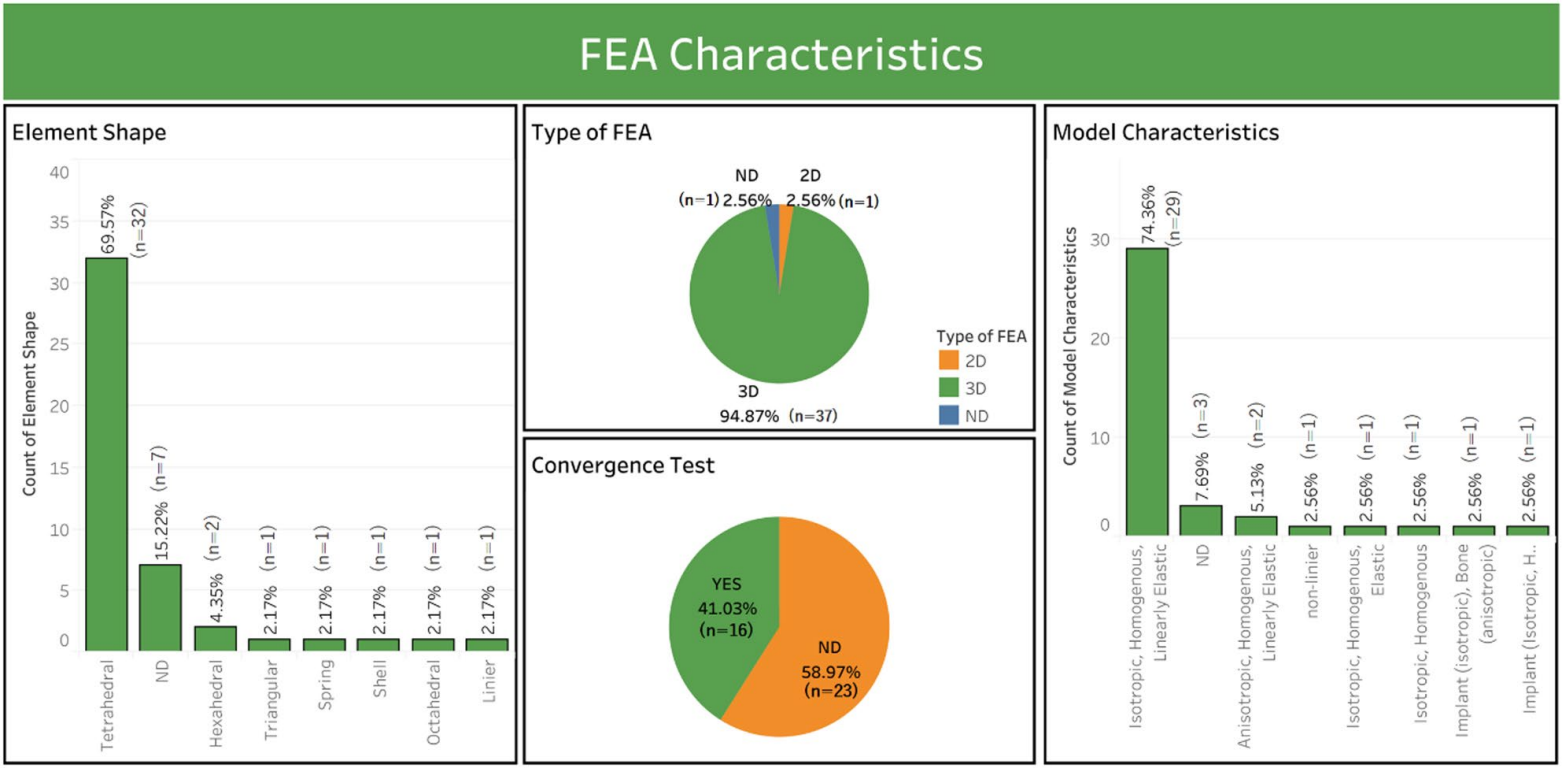
FEA characteristics of the study

**Fig. 7:**
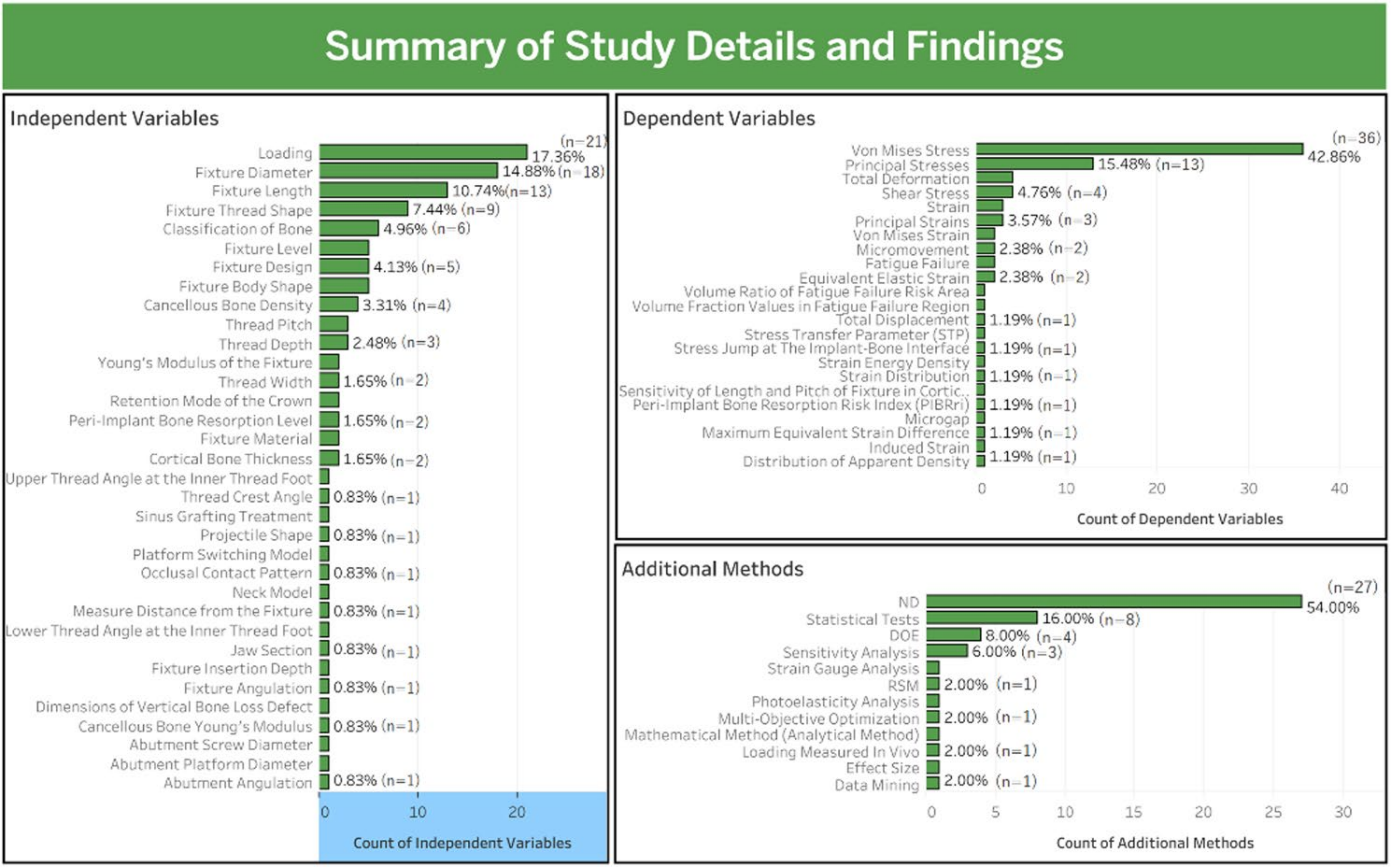
Summary of study details and findings

**Fig. 8:**
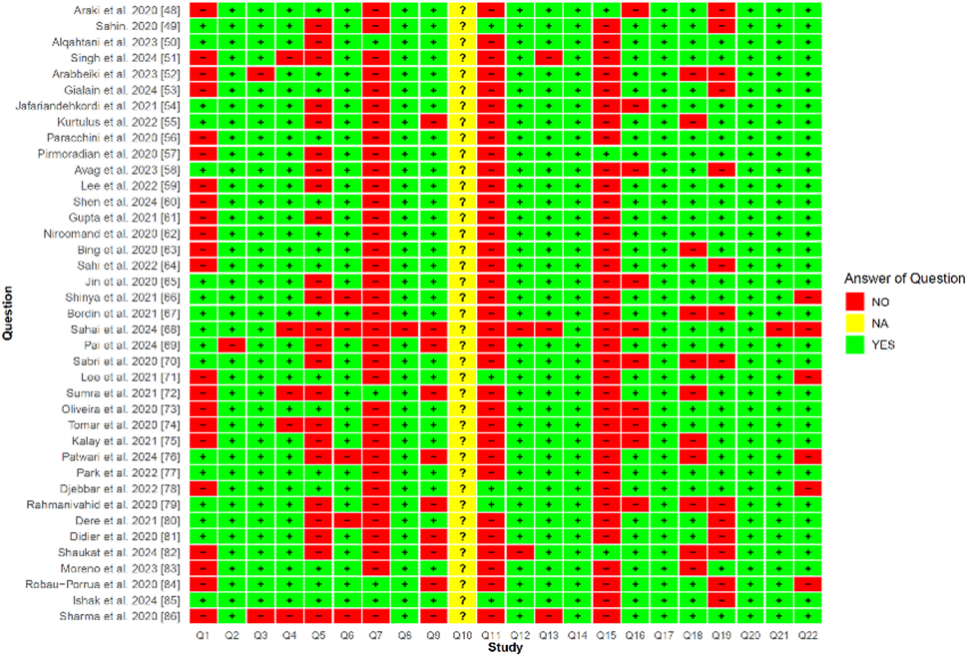
Question-wise risk of bias assessment

This section presents the data extraction results from 39 studies that met the inclusion criteria. These studies were classified based on bone characteristics, fixture characteristics, fixture dimensional, loading characteristics, FEA characteristics, and study details and findings. These categories make it easier to evaluate the influence of fixture design parameters and study data, such as thread design, diameter, and length, on posterior dental implants’ stress and strain distribution. The extracted data are presented in Tables 4, 5, 6, 7, 8 and 9 and visualized in Figs. 2, 3, 4, 5, 6, 7 and 8 according to these categories.

**Table 4:** Bone characteristics data

Author, Year	Fixture Placement Site	Classification of Bone	Bone Layers	CT Scan
Araki et al. 2020 [48]	Mandibular molar	ND	C + T	NO
Sahin. 2020 [49]	1 st mandibular and maxillary premolar	Cortical mandibula = D2 (Misch), cortical maksila = D3 (Misch)	C + T	YES
Alqahtani et al. 2023 [50]	1 st maxillary molar	D4 (Misch)	C + T	YES
Singh et al. 2024 [51]	Posterior maxilla	Cancellous Bone D4	C + T	NO
Arabbeiki et al. 2023 [52]	1 st mandibular molar	B/2 ()	C + T	NO
Gialain et al. 2024 [53]	2nd mandibular molar	Type III according to the alveolar process height (Juodzbalys and Kubilius), type I according to the alveolar process width (Juodzbalys and Kubilius)	C + T	NO
Jafariandehkordi et al. 2021 [54]	2nd mandibular premolar	ND	C + T	YES
Kurtuluş et al. 2022 [55]	Mandibular premolar	D1, D2, D3, D4 (Misch)	C + T	YES
Paracchini et al. 2020 [56]	1 st mandibular premolar	ND	C + T	NO
Pirmoradian et al. 2020 [57]	1 st mandibular molar	ND	C + T	NO
Avağ et al. 2023 [58]	1 st mandibular molar	ND	C + T	YES
Lee et al. 2022 [59]	1 st mandibular molar	ND	C + T	NO
Shen et al. 2024 [60]	1 st mandibular molar	Cancellous bone type I-IV (Rho and colleagues)	C + T	NO
Gupta et al. 2021 [61]	1 st mandibular molar	Type II (Lekholm and Zarb)	C + T	NO
Niroomand et al. 2020 [62]	1 st mandibular molar	B/2 (Lekholm and Zarb)	C + T	NO
Bing et al. 2020 [63]	1 st mandibular molar	ND	C + T	NO
Sahi et al. 2022 [64]	1 st mandibular molar	Cancellous bone density D2, D3	C + T	NO
Jin et al. 2020 [65]	Mandibular premolar	ND	C + T	YES
Shinya et al. 2021 [66]	1 st mandibular molar	ND	C + T	YES
Bordin et al. 2021 [67]	1 st maxillary molar	Type III, IV (Lekholm and Zarb)	C + T	YES
Sahai et al. 2024 [68]	Posterior mandible	ND	C + T	YES
Pai et al. 2024 [69]	1 st mandibular molar	ND	C	YES
Sabri et al. 2020 [70]	Anterior and posterior maxilla	Anterior (Tipe III/D3); Posterior (Tipe IV/D4)	C + T	YES
Lee et al. 2021 [71]	Mandibular molar	ND	C + T	NO
Sumra et al. 2021 [72]	1 st maxillary molar	D4 (Misch)	C + T	NO
Oliveira et al. 2020 [73]	1 st maxillary premolar	ND	C + T	NO
Tomar et al. 2020 [74]	1 st maxillary molar	D4/Type IV (Lekholm)	C + T	NO
Kalay et al. 2021 [75]	Molar	ND	C + T	NO
Patwari et al. 2024 [76]	1 st mandibular molar	ND	C + T	YES
Park et al. 2022 [77]	Mandibular molar	D3 and D4 (Lekholm-Zarb)	C + T	YES
Djebbar et al. 2022 [78]	Near 2nd mandibular premolar	ND	C + T	NO
Rahmanivahid et al. 2020 [79]	2nd mandibular molar	Type II	C + T	YES
Dere et al. 2021 [80]	Mandibular molar	Type 2 (Lekholm and Zarb)	C + T	YES
Didier et al. 2020 [81]	1 st and 2nd mandibular premolar	Case Study: Type IV (Lekholm)	C + T	YES
Shaukat et al. 2024 [82]	2nd mandibular molar*	ND	C + T	NO
Moreno et al. 2023 [83]	1 st mandibular molar	ND	C + T	NO
Robau-Porrua et al. 2020 [84]	Mandibular premolar	D2	C + T	NO
Ishak et al. 2024 [85]	1 st mandibular molar	Type II and III (Lekholm and Zarb)	C + T	YES
Sharma et al. 2020 [86]	1 st mandibular molar	ND	C + T	NO

*ND *Not Described, *C* Cortical, *T* Trabecular or Cancellous

*data assumed/defined based on existing information or images

**Table 5:** Fixture characteristics data

Author, year	Fixture material	Commercial product-based model	Fixture brand	Abutment-fixture connection	Fixture level	Fixture body shape	Fixture thread shape
Araki et al. 2020 [48]	cpTi, Ti-Zr	ND	ND	Two-piece*	BL, TL	Cylindrical*	ND
Sahin. 2020 [49]	Titanium, Ti-Zr	YES	Straumann	Two-piece*	BL	Cylindrical	ND
Alqahtani et al. 2023 [50]	Titanium	ND	ND	Two-piece*	BL*	Cylindrical-tapered apex	*Vs*,* Sq*,* Bt*
Singh et al. 2024 [51]	Titanium	ND	ND	Two-piece*	BL*	Tapered, step	Acme
Arabbeiki et al. 2023 [52]	Titanium	YES	Straumann	Two-piece*	TL*	Cylindrical*	*Vs*,* Sq*,* Bt*,* RBt*
Gialain et al. 2024 [53]	Ti-6Al-4 V*	YES	Straumann	Two-piece*	TL	Cylindrical*	*Tz*
Jafariandehkordi et al. 2021 [54]	Titanium	ND	ND	Two-piece*	BL*	Cylindrical*	Threadless*
Kurtuluş et al. 2022 [55]	Titanium	YES	Straumann	Two-piece*	BL	Tapered*, cylindrical-tapered apex*	*Rbt*, *VDT*
Paracchini et al. 2020 [56]	Titanium	YES	Ornaghi	Two-piece*	BL*	Cylindrical, tapered with reverse conical neck	*Vs*, nest shape
Pirmoradian et al. 2020 [57]	Titanium	ND	ND	Two-piece*	TL*	Cylindrical*	Tz*
Avağ et al. 2023 [58]	Titanium	YES	Straumann, NTA Implant	Two-piece*	BL, TL	Cylindrical*, tapered*	RBt*, plateu thread design
Lee et al. 2022 [59]	Titanium	YES	Osstem Implant	Two-piece*	BL, TL	Tapered*	ND
Shen et al. 2024 [60]	Titanium	YES	Biomet 3i	Two-piece*	TL*	Tapered*	ND
Gupta et al. 2021 [61]	Ti-Al-4 V	YES	(Nobel Biocare)	One-piece	BL*	Cylindrical-tapered apex*	*Vs*
Niroomand et al. 2020 [62]	Titanium	YES	Straumann	One-piece*	TL*	Cylindrical*	*Tz*
Bing et al. 2020 [63]	Titanium	ND	ND	Two-piece*	BL*	Cylindrical-tapered apex*	Threadless*
Sahi et al. 2022 [64]	Titanium	YES	MIS Implant, Osstem Implant	Two-piece*	BL*	Tapered, tapered*	*RBt* dual thread, *Vs* with double lead thread
Jin et al. 2020 [65]	Titanium	ND	ND	One-piece	BL*	Cylindrical	*Vs*
Shinya et al. 2021 [66]	Titanium	YES	Camlog	One-piece	BL*	Cylindrical-tapered apex*	ND
Bordin et al. 2021 [67]	Titanium	ND	ND	Two-piece*	BL*	Cylindrical-tapered apex*	*Vs*
Sahai et al. 2024 [68]	ND	ND	ND	ND	ND	ND	*Vs*,* Sq*
Pai et al. 2024 [69]	Titanium Alloy	YES	Nobel Biocare	Two-piece*	TL*	Cylindrical-tapered apex*	ND
Sabri et al. 2020 [70]	Titanium	ND	ND	Two-piece*	TL*	Cylindrical, tapered	*Vs*
Lee et al. 2021 [71]	Ti-6Al-4 V	YES	Osstem Implant	Two-piece*	BL, TL	Tapered*	ND
Sumra et al. 2021 [72]	Titanium	ND	ND	Two-piece*	BL*	Tapered	*Vs*,* Sq*,* Bt*, acme
Oliveira et al. 2020 [73]	Titanium Grade 3, Titanium Grade 4	YES	Klockner	One-piece	BL, TL	Cylindrical-tapered apex with concave tulip-shaped beck, cylindrical-tapered apex with convex cone-shaped	*Tz*
Tomar et al. 2020 [74]	Titanium	YES	Nobel Biocare	One-piece	BL*	Tapered*	Single-threaded, double-threaded, triple-threaded, asymmetric threaded
Kalay et al. 2021 [75]	Ti-6Al-4 V	YES	Nobel Biocare	One-piece	TL*	Tapered*	*Vs*,* Sq*,* Bt*,* RBt*
Patwari et al. 2024 [76]	Titanium Alloy	ND	ND	Two-piece*	BL*	Cylindrical*	*Vs*,* Sq*,* Bt*,* RBt*
Park et al. 2022 [77]	Ti-6Al-4 V	YES	Osstem Implant	Two-piece*	BL, TL	Tapered*	ND
Djebbar et al. 2022 [78]	Ti-6Al-4 V	ND	ND	Two-piece*	TL*	Cylindrical*	*Vs*,* Sq*,* Tz*,* Rd*
Rahmanivahid et al. 2020 [79]	Ti-6Al-4 V	YES	(Megagen), (Nobel, (Zimmer	Two-piece*	BL*	Cylindrical-tapered apex	*Vs*, variable fins
Dere et al. 2021 [80]	Titanium	ND	ND	Two-piece*	BL*	Cylindrical, tapered	*Vs*
Didier et al. 2020 [81]	Ti-6Al-4 V, Ti-Nb	ND	ND	Two-piece*	BL*	Cylindrical, tapered	ND
Shaukat et al. 2024 [82]	Ti-6Al-4 V	ND	ND	Two-piece*	BL*	Tapered	*Bt*,* Tz*
Moreno et al. 2023 [83]	Titanium Grade 4	YES	S.I.N Implant	Two-piece*	BL*	Cylindrical-tapered apex*	ND
Robau-Porrua et al. 2020 [84]	Ti-6Al-4 V	ND	ND	One-piece*	TL*	Tapered	*Sq*
Ishak et al. 2024 [85]	Ti-6Al-4 V	YES	Alpa Bio Tec	Two-piece*	BL	Tapered*	*Vs*
Sharma et al. 2020 [86]	Titanium	YES	MIS Implant	Two-piece*	BL*	Cylindrical, tapered	*Vs*,* Bt*,* RBt*

*ND *Not Described, *TL *Tissue level, *BL* Bone level, *Vs* V-shape, *Bt* Buttress, *RBt* Inverted buttress, *Sq* Square, *Tz* Trapezoid, *Rd* Round, *VDT* Variable double thread

*data assumed/defined based on existing information or images

**Table 6:** Fixture dimensional data

Author, Year	Diameter (mm)	Length (mm)	Thread Width (mm)	Thread Depth (mm)	Thread Pitch (mm)
Araki et al. 2020 [48]	4.1	4.0, 6.0, 8.0, 10.0	ND	ND	ND
Sahin. 2020 [49]	3.3, 4.1	10.0	ND	ND	ND
Alqahtani et al. 2023 [50]	4.0, 5.0, 6.0	6.0	0.8	0.4	ND
Singh et al. 2024 [51]	3.75	6.0, 10.0	ND	ND	Body = 0.8; Microthread = 0.2
Arabbeiki et al. 2023 [52]	4.1	13.0	0.1–0.3	0.15–0.35	0.55–1.05
Gialain et al. 2024 [53]	4.1, 4.8, 4.8-wide-neck	4.0, 6.0, 8.0, 10.0	ND	0.35	0.8, 1.2
Jafariandehkordi et al. 2021 [54]	3.6, 4.0, 4.4	9.0, 10.0, 11.0	ND	ND	ND
Kurtuluş et al. 2022 [55]	4.0, 4.1	10.0	ND	ND	ND
Paracchini et al. 2020 [56]	4.0	11.0	ND	0.35, 0.5	0.6
Pirmoradian et al. 2020 [57]	4.1	8.5, 10.0, 11.5, 13.0	0.2	0.35	0.6, 0.7, 0.8, 1.0
Avağ et al. 2023 [58]	4.0, 4.1	6.0	ND	ND	ND
Lee et al. 2022 [59]	3.5, 4.0, 4.5	10.0	ND	ND	ND
Shen et al. 2024 [60]	4.0, 5.0	13.0	ND	ND	ND
Gupta et al. 2021 [61]	3.75, 4.3, 5.0	11.5	ND	ND	ND
Niroomand et al. 2020 [62]	3.4, 4.1, 4.8	10.0, 13.0, 16.0	0.1, 0.2, 0.3	0.25, 0.35, 0.45	0.75, 1.05, 1.35
Bing et al. 2020 [63]	3.5, 4.0, 5.0	13.0	ND	ND	ND
Sahi et al. 2022 [64]	4.0, 4.2	10.0	ND	Body = 0.5, 0.6; Microthread = 0.2; Microrings = 0.1	Body = 0.8, 2.4; Microthread = 0.8; Microrings = 0.3
Jin et al. 2020 [65]	4.0	11.5	ND	ND	Body = 1.2; Microthread = 0.25
Shinya et al. 2021 [66]	3.8, 4.3, 6.0	9.0, 11.0, 13.0, 16.0	ND	ND	ND
Bordin et al. 2021 [67]	4.0	7.0, 13.0	ND	ND	0.55
Sahai et al. 2024 [68]	3.5, 4.3	10.0, 11.5	ND	ND	ND
Pai et al. 2024 [69]	3.5, 4.3, 5.0, 6.0	13.6	ND	ND	0.64, 0.71, 0.75, 0.79
Sabri et al. 2020 [70]	4.0	10.0	ND	0.25, 0.35, 0.45	0.8
Lee et al. 2021 [71]	3.5, 4.0, 4.5	10.0	ND	ND	ND
Sumra et al. 2021 [72]	4.0, 5.0, 6.0	5.0	ND	Body = 0.4 Microthread = 0.1	Body = 0.5 Microthread = 0.2
Oliveira et al. 2020 [73]	4.0	10.0	ND	ND	Body = 2.2; Microthread = 0.05
Tomar et al. 2020 [74]	5.0	7.0	ND	All body = 0.5; Coronal in Asymmetrical = 0.25	Body = 0.8, 1.6, 2.4; Coronal in Asymmetric = 0.4
Kalay et al. 2021 [75]	3.5	13.0	0.2	0.25	0.6
Patwari et al. 2024 [76]	4.0	10.0	ND	0.4	ND
Park et al. 2022 [77]	4.0, 4.5, 5.0	7.0, 10.0, 13.0	ND	ND	ND
Djebbar et al. 2022 [78]	3.8	14.0	ND	0.48	1.2
Rahmanivahid et al. 2020 [79]	5.0	10.5	ND	Body = 0.4; Depth Fin Edges = 0.5, 0.4, 0.3, 0.2, 0.1	0.8
Dere et al. 2021 [80]	3.75	10.0	ND	ND	0.6
Didier et al. 2020 [81]	3.0, 4.0	10.0, 15.0	ND	ND	ND
Shaukat et al. 2024 [82]	4.0	12.0	ND	ND	0.8
Moreno et al. 2023 [83]	4.0, 6.0	5.0	ND	ND	ND
Robau-Porrua et al. 2020 [84]	3.8, 4.5	10.0, 13.0	ND	ND	1.2
Ishak et al. 2024 [85]	3.75	4.0, 6.0, 10.0, 13.0, 15.0	ND	ND	ND
Sharma et al. 2020 [86]	3.75	11.5	ND	0.42	1.2

*ND *Not Described

**Table 7:** Loading characteristics data

Author, year	Load type	Axial load	Lateral load	Oblique load	Area of load application	Additional loading
Araki et al. 2020 [48]	Static	ND	ND	100 N (30°)	The occlusal plane of the superstructure from the buccal view	ND
Sahin. 2020 [49]	Static, dynamic	100 N	ND	100 N (45°)	Axial (on the mesial fossa and palatal cusp for the maxilla and the distal fossa and buccal cusp for the mandible); Oblique (on the palatal slope of the maxillary buccal cusp in the palatal-to-buccal direction and on the mandibular buccal cusp in the buccal-to-lingual direction)	Dynamic loading: 10⁷ cycles, corresponding to a 30-year lifespan
Alqahtani et al. 2023 [50]	Static (immediate)	100 N	ND	100 N (45°)	At the central fossa	ND
Singh et al. 2024 [51]	Static (immediate)	100 N	ND	100 N (45°)	ND	ND
Arabbeiki et al. 2023 [52]	Static	ND	ND	100 N (45°)	Center of the ceramic crown with buccolingual force	ND
Gialain et al. 2024 [53]	Static	200 N	ND	ND	3 occlusal contact areas and 6 occlusal contact areas of the crown	ND
Jafariandehkordi et al. 2021 [54]	Static pressure load	1.117 MPa	ND	ND	The upper surface of the dental crown	ND
Kurtuluş et al. 2022 [55]	Static	ND	ND	200 N (30°)	The occlusal of the abutment	ND
Paracchini et al. 2020 [56]	Static	100 N	ND	223.6 N (25°)	The long axis of the fixture	ND
Pirmoradian et al. 2020 [57]	Static	180 N	ND	180 N (45°)	The middle point at the center of the superstructure	ND
Avağ et al. 2023 [58]	Static	150 N	ND	50 N (45°)	Axial (central fossa of the crown); Oblique (mesiobuccal cusp of the crown)	ND
Lee et al. 2022 [59]	Static	200 N	ND	100 N (45°)	Vertical Loading (60 nodes on 3 buccal cusps and 3 fossae); Oblique Loading (30 nodes on 3 buccal cusps)	Tightening torque of 32 Ncm applied to the abutment screw
Shen et al. 2024 [60]	Static	300 N	100 N	300 N (30°)	Axial (occlusal center); Oblique (surface of the buccal cusp); Lateral (buccal cusp)	Preload torque of 32 Ncm at the abutment screw
Gupta et al. 2021 [61]	Static	ND	ND	118.2 N (75°)	Center of the upper surface of the abutment	ND
Niroomand et al. 2020 [62]	Static	ND	ND	100 N (45°)	The center of the prosthetic crown with buccolingual force	ND
Bing et al. 2020 [63]	Static	ND	ND	177.6 N (3°), 46.5 N (37.2°), 32.4 (21.5°)	The top surface of the abutment	ND
Sahi et al. 2022 [64]	Static	200 N	ND	80 N (30°)	Axial (central groove, mesio-buccal, disto-buccal cusp tips 1.5 mm away from the central groove, distal and mesial marginal ridges each 1 mm from the distal and mesial surfaces); Oblique (central groove on the buccal inclines of mesio-buccal and disto-buccal cusps)	ND
Jin et al. 2020 [65]	Static	ND	ND	150 N (45°)	Top center point of the abutment from buccal to lingual	ND
Shinya et al. 2021 [66]	Static	ND	ND	50 N (45°)	Outer inclination of the buccal cusp from the buccal side in the lingual direction	ND
Bordin et al. 2021 [67]	Static	300 N	ND	ND	The occlusal surface of the crown	ND
Sahai et al. 2024 [68]	Static	Load applied but magnitude not reported	Load applied (nonaxial buccolingual and mesiodistal load) but magnitude not reported	ND	ND	ND
Pai et al. 2024 [69]	Static	100 N	Buccolingual Load = 50 N; Mesiodistal Load = 50 N	ND	Implant top surface	ND
Sabri et al. 2020 [70]	Static	250 N	ND	ND	Center of the supra structure of the implant	ND
Lee et al. 2021 [71]	Static, dynamic	200 N	ND	100 N (45°)	Axial (60 nodes on the three cusps at buccal and three fossae); Oblique (30 nodes on the three cusps at buccal)	Preload/torque loading at the abutment screw = 32 Ncm; Dynamic loading = 10⁷ cycles
Sumra et al. 2021 [72]	Static	100 N	ND	100 N (45°)	Crown	ND
Oliveira et al. 2020 [73]	Static	ND	ND	150 N (30°)	Central fossa of the restoration	ND
Tomar et al. 2020 [74]	Static	ND	ND	100 N (15°)	Mid-center of the abutment of the one-piece system implant, load was applied in the buccopalatal direction	ND
Kalay et al. 2021 [75]	Static	150 N, 200 N, 250 N, 300 N	ND	ND	The top of all implant models	ND
Patwari et al. 2024 [76]	Static	100 N	ND	100 N (45°)	On the abutment in the buccolingual plane	ND
Park et al. 2022 [77]	Static, time-dependent	50 N (time-dependent after 150 days due to the remodeling process)	ND	200 N (30°) (after 150 days of remodeling)	50 nodes at three fossae and three cusps crown	Preload/torque loading at the abutment screw = 32 Ncm
Djebbar et al. 2022 [78]	Impact	ND	ND	ND	Towards the bone along the Z-axis (impact zone and bone)	Tightening torque of 35 Ncm at both the abutment–implant and implant–bone interfaces; Mass of the hemispherical projectile = 3.5 g; Mass of the conical projectile = 8.0 g; Projectile velocity = 10 m/s
Rahmanivahid et al. 2020 [79]	Static, cyclic, displacement	150 N (static); 50 N (cyclic)	50 N, 75 N	ND	Implant model with lateral loading is combined horizontal loading in the buccal-lingual plane	Pull out displacement and pull in displacement = 0.01 mm
Dere et al. 2021 [80]	Static insertion torque	ND	ND	ND	The top of the implants	clockwise torque = 450 Nmm
Didier et al. 2020 [81]	Static	120 N	ND	ND	On the top surface of the abutment	ND
Shaukat et al. 2024 [82]	Static	ND	ND	ND	Five distinct distributive points on the occlusal surface of the crown	Combined loading up to 500 N was applied (details not reported)
Moreno et al. 2023 [83]	Static	200 N	ND	200 N (30°)	Mesiobuccal cusp of the crown	ND
Robau-Porrua et al. 2020 [84]	Static (Immediate)	114.6 N	Bucco-lingual Load = 17.1 N; Mesial-distal Load = 23.4 N	ND	Surface of the coronal area of the dental implants	ND
Ishak et al. 2024 [85]	Dynamic	300 N	ND	ND	The upper surface of the prosthesis	Pre-tension force scenario 20 N onto the external surface of the abutment screw
Sharma et al. 2020 [86]	Static	100 N	Buccolingual Load = 50 N	ND	ND	ND

*ND* Not Described

**Table 8:** FEA characteristics data

Author, year	Type of fea	Element shape	Convergence test	Model characteristics	Fea software
Araki et al. 2020 [48]	3D	Tetrahedral	YES	ND	ND
Sahin. 2020 [49]	3D	8-node tetrahedral	ND	Isotropic, homogenous, linearly elastic	ND
Alqahtani et al. 2023 [50]	3D	10-node tetrahedral, octahedral with 3 DOF per node	ND	Anisotropic, homogenous, linearly elastic	Ansys R v.18.1
Singh et al. 2024 [51]	2D	ND	ND	Isotropic, homogenous, linearly elastic	ANSYS classic, version 11
Arabbeiki et al. 2023 [52]	3D	Quadratic tetrahedral, hexahedral	YES	Isotropic, homogenous	ND
Gialain et al. 2024 [53]	3D	Tetrahedral	YES	Isotropic, homogenous, linearly elastic	ND
Jafariandehkordi et al. 2021 [54]	3D	10-node quadratic tetrahedral	ND	Isotropic, homogenous, linearly elastic	COMSOL Multiphysics 5.4
Kurtuluş et al. 2022 [55]	3D	Quadratic tetrahedral	ND	Isotropic, homogenous, linearly elastic	Ansys R v.18.1
Paracchini et al. 2020 [56]	3D	Quadratic tetrahedral, quadratic triangular shell, quadratic spring	YES	Isotropic, homogenous, linearly elastic	Ansys WB 2.0 Framework, Version 12.0.1, 2009
Pirmoradian et al. 2020 [57]	3D	10-node quadratic tetrahedral	ND	Isotropic, homogenous, linearly elastic	ABAQUS (6.14.2)
Avağ et al. 2023 [58]	3D	ND	ND	Isotropic, homogenous, linearly elastic	Algor Software
Lee et al. 2022 [59]	3D	Tetrahedral	ND	Isotropic, homogenous, linearly elastic	ABAQUS 6.14
Shen et al. 2024 [60]	3D	10-node SOLID187	YES	Isotropic, homogenous, elastic	Ansys Software Version 19
Gupta et al. 2021 [61]	3D	4-node linear tetrahedral	ND	Isotropic, homogenous, linearly elastic	ANSYS Workbench version 18.1
Niroomand et al. 2020 [62]	3D	Quadratic tetrahedral, hexahedral	YES	Isotropic, homogenous, linearly elastic	ANSYS Workbench 19.1
Bing et al. 2020 [63]	3D	Tetrahedral	YES	Isotropic, homogenous, linearly elastic	Mechanical Finder Ver. 8.0 Extended Edition
Sahi et al. 2022 [64]	3D	Tetrahedral	YES	Isotropic, homogenous, linearly elastic	ND
Jin et al. 2020 [65]	3D	Tetrahedral	ND	Isotropic, homogenous, linearly elastic	Abaqus2016
Shinya et al. 2021 [66]	3D	4-node tetrahedral	ND	Non-linier	Mechanical Finder ver. 5.1; VX-CAD/CAM
Bordin et al. 2021 [67]	3D	Tetrahedral	YES	Isotropic, homogenous, linearly elastic	Ansys Workbench
Sahai et al. 2024 [68]	ND	ND	ND	ND	ANSYS Workbench 17.0
Pai et al. 2024 [69]	3D	10-node tetrahedral with 3 DOF per node	ND	Isotropic, homogenous, linearly elastic	ANSYS Workbench v11.0
Sabri et al. 2020 [70]	3D	ND	ND	Isotropic, homogenous, linearly elastic	ANSYS Workbench
Lee et al. 2021 [71]	3D	Tetrahedral	YES	Isotropic, homogenous, linearly elastic	ABAQUS 6.14
Sumra et al. 2021 [72]	3D	ND	ND	Anisotropic, homogenous, linearly elastic	ANSYS 12.1
Oliveira et al. 2020 [73]	3D	ND	YES	Isotropic, homogenous, linearly elastic	Ansys 2020 R1
Tomar et al. 2020 [74]	3D	10-node tetrahedral with 4 DOF per node	ND	Isotropic, homogenous, linearly elastic	ANSYS 13.0
Kalay et al. 2021 [75]	3D	4-node tetrahedral with 4 faces	ND	Isotropic, homogenous, linearly elastic	ANSYS 16.1
Patwari et al. 2024 [76]	3D	10-node tetrahedral with 3 DOF per node	ND	Isotropic, homogenous, linearly elastic	ANSYS version 14.5
Park et al. 2022 [77]	3D	4-node tetrahedral	YES	Isotropic, homogenous, linearly elastic	ABAQUS 6.14
Djebbar et al. 2022 [78]	3D	10-node tetrahedral	YES	Isotropic, homogenous, linearly elastic	Abaqus program 6.13
Rahmanivahid et al. 2020 [79]	3D	ND	ND	Isotropic, homogenous, linearly elastic	Abaqus software
Dere et al. 2021 [80]	3D	10-node tetrahedral	ND	Isotropic, homogenous, linearly elastic	ND
Didier et al. 2020 [81]	3D	Quadratic tetrahedral	ND	ND	ND
Shaukat et al. 2024 [82]	3D	Linear, triangular, and tetrahedral	ND	Isotropic, homogenous, linearly elastic	Ansys software
Moreno et al. 2023 [83]	3D	Tetrahedral	YES	Isotropic, homogenous, linearly elastic	ANSYS Workbench 15.0
Robau-Porrua et al. 2020 [84]	3D	10-node tetrahedral	YES	Implant (Isotropic, Homogenous, Linearly Elastic), Bone (Anisotropic, non-Homogenous, Linearly Elastic	Abaqus/CAE
Ishak et al. 2024 [85]	3D	4-node tetrahedral with 3 DOF	YES	Implant (isotropic), Bone (anisotropic)	ANSYS software platform
Sharma et al. 2020 [86]	3D	10-node tetrahedral	ND	Isotropic, homogenous, linearly elastic	ANSYS 18.1

*ND* Not Desctibed, *DOF* Degree of Freedom

**Table 9:** Study details and findings data

Author, year	Additional methods	Part of the simulation	Independent variables	Dependent variables	Boundary condition	Main findings
Araki et al. 2020 [48]	ND	Superstructure, abutment screw, fixture, cortical bone, cancellous bone, and mandibular incisive canal	Fixture level, fixture length and fixture material	Maximum principal stress and von Mises stress	Full osseointegration at the implant-bone interface. Implant components have a friction coefficient (µ = 0.3). Mesial and distal sides of the alveolar bone are fixed.	Tissue-level (tl) fixture exhibit lower stress than bone-level (bl) fixture in the implant and cortical bone. Fixture length is inversely proportional to stress, but fixture level has a greater influence.
Sahin. 2020 [49]	ND	Superstructure, crown framework, abutment screw, abutment, fixture, cortical bone, and cancellous bone	Fixture diameter, fixture material, jaw section, loading	Maximum principal stress, minimum principal stress, von Mises stress, and fatigue failure	Full osseointegration at the implant-bone interface with bonded contact. Inferior edge of the mandible, superior edge of the maxilla, and lateral jaw regions are fixed. Soft tissues are not modeled.	Narrow-diameter fixture (3.3 mm) produce higher stress than standard-diameter fixture (4.1 mm), with stress reduction under axial and oblique loads as diameter increases. Smaller diameters pose higher clinical risks.
Alqahtani et al. 2023 [50]	Statistical Tests: Shapiro-Wilk W Test, Levene’s Test, T-Student Test ANOVA with Tukey’s Post Hoc Test Kruskal-Wallis Test with Dunn’s Post Hoc Test	Crown, crown framework, cement layer, abutment, fixture, cortical bone, and cancellous bone	Fixture thread shape, fixture diameter, loading	Von Mises stress, strain, and micromovement	Immediate loading protocol applied with a friction coefficient (µ = 0.3) at the implant-bone interface. Bone base is fixed.	A 6-mm diameter with square threads yields the lowest stress, strain, and micromovement compared to 4-mm diameter or other thread shapes, particularly under oblique loads, which increase stress. A 6-mm diameter short fixture with square threads is recommended for atrophic posterior maxilla.
Singh et al. 2024 [51]	ND	Abutment, abutment screw, fixture, cortical bone, and cancellous bone	Fixture body shape, fixture length, and loading	Total deformation, von Mises stress, and von Mises strain	Implant-bone interface has a friction coefficient (µ = 0.6).	Step fixture produce lower stress and strain than tapered fixture. A 10-mm length outperforms a 6-mm length. Crestal microthreads reduce cortical stress. A step fixture with a 3.75-mm diameter and 10-mm length is recommended for immediate loading in low-density bone with optimal height.
Arabbeiki et al. 2023 [52]	DOE: Latin Hypercube Sampling (LHS); Data Mining: Decision Tree; Statistical Tests: Receiver Operating Characteristic (ROC), Area Under the Curve (AUC)	Crown, abutment, fixture, cortical bone, and cancellous bone	Thread depth, thread width, thread pitch, upper thread angle at the inner thread foot, lower thread angle at the inner thread foot	Von Mises stress, maximum principal stress, and shear stress	Mesial and distal bone surfaces are fixed. Implant-bone interface has full bonding without slippage or separation.	Thread depth (0.25 < td < 0.34) and thread width (0.25 ≤ tw ≤ 0.28) are more significant than thread pitch (0.8 ≤ tp ≤ 0.88) in peri-implant bone remodeling. V-shaped threads with upper thread angle (66.2 ≤ α ≤ 80) and lower thread angle (β ≤ 65.4) provide better stability than other thread designs.
Gialain et al. 2024 [53]	ND	Crown, abutment, fixture, cortical bone, cancellous bone, and mandibular canal	Fixture diameter, fixture length, bone classification, and occlusal contact pattern	Strain energy density and peri-implant bone resorption risk index (pibrri)	Proximal bone nodes are fixed to prevent movement. Full osseointegration at the implant-bone interface. Screw preload is neglected.	A 4.8-mm diameter with wide-neck and broad load distribution (6 occlusal contact areas) reduces bone resorption by 35–40%. A 6-mm length decreases mechanical stimulation by 18–26%. Short, large-diameter fixture are safe for atrophic mandibles.
Jafariandehkordi et al. 2021 [54]	Sensitivity analysis	Premolar enamel, premolar dentine, crown, abutment screw, abutment, fixture, cortical bone, and cancellous bone	Fixture diameter and fixture length	Maximum equivalent strain difference	Left and right trabecular mandibular surfaces are fixed. Internal and external dental implant components are bonded. Full osseointegration at the implant-mandible interface.	Diameter (optimal: 4 mm) is more sensitive to strain than length (optimal: 11 mm). Design optimization reduces maximum strain by 50.2%.
Kurtuluş et al. 2022 [55]	ND	Abutment screw, abutment, fixture, cortical bone, and cancellous bone	Fixture design, abutment angulation, and bone classification	Von Mises stress, maximum principal stress, and minimum principal stress	Full osseointegration at the implant-bone interface. Bone base is fixed in x, y, z axes. Implant-abutment interface has a friction coefficient (µ = 0.3). All other contacts are rigidly bonded.	Blt implants produce lower stress than blx, but blx is more stable. A 17° abutment angulation increases stress.
Paracchini et al. 2020 [56]	ND	Fixture, cortical bone, and cancellous bone	Fixture design and loading	Von Mises stress and equivalent elastic strain	Full osseointegration at the implant-bone interface. Cortical implant-bone interface has a friction coefficient (µ = 0.65), cancellous implant-bone interface (µ = 0.77). Bone base is fixed to prevent rotation or displacement. Nonlinear contact is applied at cortical and cancellous implant-bone interfaces.	Conical fixture with reverse conical necks and “nest shape” threads reduce cortical stress compared to cylindrical fixture with v-shaped threads.
Pirmoradian et al. 2020 [57]	Photoelasticity analysis, sensitivity analysis	Superstructure, abutment, fixture, cortical bone, and cancellous bone	Fixture length, thread pitch, and loading	Von Mises stress, sensitivity of length and pitch in cortical and cancellous bone	Implants are rigidly anchored with a rough, screwed surface at the mandibular bone interface. Abutment-prosthesis interface is rigidly bonded. Mesial and distal cross-sectional planes lack cortical bone coverage.	A 13-mm length and 0.7-mm pitch yield the lowest stress. Fixture length is more sensitive than pitch. Optimal dimensions from sensitivity analysis are 11.5-mm length and 0.8-mm pitch.
Avağ et al. 2023 [58]	ND	Crown, crown framework, abutment, abutment screw, and fixture, half of mandible jaw (cortical bone and cancellous bone)	Fixture level and loading	Von Mises stress, maximum principal stress, and minimum principal stress	Full osseointegration at the implant-bone interface. Crown, abutment, and abutment screw are considered a single unit. Degrees of freedom at the condyle, coronoid process, and mandibular angle are fixed.	Tl fixture distribute stress more evenly than bl fixture. Short bl fixture produce high stress (342.77 MPa) under oblique loading.
Lee et al. 2022 [59]	Statistical tests: General linear model, Multi-Level Interactions Post-Hoc Tukey Test; Effect Size: Partial Eta Squared (η²)	Crown, cement layer, abutment, abutment screw, fixture, cortical bone, cancellous bone, nerve canal, and cylindrical part virtual near the bone fixture interface	Fixture diameter, fixture level, cancellous bone density, and loading	Von Mises stress, maximum principal strain, minimum principal strain, volume ratio of fatigue failure risk area	Full osseointegration at the implant-bone interface. Implant-abutment interface has a friction coefficient (µ = 0.16), abutment-screw (µ = 0.441), cement-abutment (µ = 0.25). Mesial and distal surfaces of cortical and cancellous bone are fixed in x, y, and z axes.	Tl fixture outperform bl fixture in stress distribution at the fixture and abutment. Small-diameter fixture increase stress concentration, but connection type has a greater influence.
Shen et al. 2024 [60]	ND	Crown, abutment screw, abutment, fixture, cortical bone, and cancellous bone	Fixture diameter, abutment platform diameter, abutment screw diameter, cancellous bone density, and loading	Von Mises stress, principal stress	Full osseointegration with bonded contact at the alveolar bone-implant interface. Alveil bone has uniform thickness. Cement and metal coping prostheses are neglected. Chewing patterns, occlusal forces, and alveolar bone response are simplified.	Wider fixture diameters reduce stress on bone and fixture, but larger abutment screw diameters increase stress on the screw and fixture.
Gupta et al. 2021 [61]	ND	Abutment-fixture, cortical bone, and cancellous bone	Fixture diameter, dimensions of vertical bone loss defect	Von Mises stress	Full osseointegration at the implant-bone interface. Vertical bone loss is modeled as an isosceles triangle with a 45° angle surrounding the implant. Mesial and distal bone sides are fixed. Soft tissues are not modeled.	The optimal fixture diameter for vertical bone loss due to peri-implantitis is 4.3 mm. When bone loss exceeds 10%, stress shifts from cortical to cancellous bone.
Niroomand et al. 2020 [62]	DOE: Central Composite Design (CCD), RSM, Sensitivity Analysis, Statistical Test: Main Effect Plot	Prosthetic crown, abutment-fixture, cortical bone, and cancellous bone	Fixture diameter, fixture length, thread depth, thread width, thread pitch, and thread crest angle	Von Mises stress, maximum total deformation	Full osseointegration at the implant-bone interface without separation or displacement. The mesial and distal mandibular sides are fixed.	Fixture body parameters influence stability more than threads. A 4.77-mm diameter effectively reduces cortical stress, and a 12.72-mm length minimizes cancellous deformation. Thread width (0.18 mm), depth (0.27 mm), angle (33.48°), and pitch (0.79 mm) are critical for optimal stress distribution and stability.
Bing et al. 2020 [63]	Loading Measured In Vivo Using a 3D Piezoelectric Force Transducer	Abutment screw, abutment, fixture, cortical bone, and cancellous bone	Fixture diameter, peri-implant bone resorption level, and loading	Von Mises stress	Mesial and distal bone surfaces are fixed. Full osseointegration at the implant-bone interface. Implant components are bonded.	A 2-mm bone resorption increases von mises stress on the fixture and abutment screw. Small diameters increase fracture risk due to higher stress. Large-angle loads cause uneven stress distribution with peak stress above peri-implant bone.
Sahi et al. 2022 [64]	ND	Crown, crown framework, cement layer, abutment screw, abutment, fixture, cortical bone, and cancellous bone	Fixture design, bone classification, and loading	Von Mises stress	Full osseointegration at the implant-bone and abutment screw-implant interfaces. Inferior bone boundary is fixed.	Mis seven implants with reverse buttress threads produce lower von Mises stress than Osstem TS III V-shaped threads in d2/d3 bone. Highest stress occurs at the implant-abutment interface, screw neck, and cervical bone. Cortical bone stress exceeds cancellous bone stress.
Jin et al. 2020 [65]	ND	Abutment-fixture, cortical bone, and cancellous bone	Neck model, peri-implant bone resorption level	Von Mises stress, maximum principal stress, minimum principal stress, shear stress	Full osseointegration at the implant-bone interface. Mesial and distal boundary nodes are fixed in all directions.	T-neck microthread design reduces shear stress and marginal bone resorption, but effectiveness decreases when resorption ≥ 0.75 mm.
Shinya et al. 2021 [66]	ND	Prosthetic device, abutment-fixture, and mandible jaw (cortical bone and cancellous bone)	Fixture diameter and fixture length	Von Mises stress	The inferior mandibular boundary is fixed.	A 6-mm diameter reduces peri-implant stress by approximately 50% compared to 3.8 mm, with a more significant effect than increasing fixture length from 9 to 16 mm.
Bordin et al. 2021 [67]	ND	Crown, crown framework, cement layer, abutment screw, abutment, fixture, cortical bone, cancellous bone, and grafted sinus bone	Fixture length, sinus grafting treatment, retention mode of the crown	Von Mises stress, maximum principal stress, minimum principal stress, and shear stress	Bone graft achieves full osseointegration after six months of healing. Lateral bone sides are bonded. Full osseointegration at the implant-bone interface without friction coefficient.	A 13-mm fixture with sinus grafting reduces shear stress by 35.75% (cortical) and 51% (trabecular) compared to a 7-mm fixture. This combination is recommended for posterior maxillary rehabilitation.
Sahai et al. 2024 [68]	Statistical Test: Chi-Square Test	Fixture, cortical bone, and cancellous bone	Fixture diameter, fixture length, fixture thread shape, and loading	Von Mises stress, strain	Not specified	Square threads produce lower stress concentrations than V-shaped threads at the fixture and cancellous bone surfaces, consistent across various axial and non-axial loads.
Pai et al. 2024 [69]	ND	Fixture, cortical bone, and cancellous bone	Fixture diameter and loading	Von Mises stress	Distal bone end is fixed in all three directions. Inferior support is removed for bending. Full osseointegration at the implant-bone interface.	A 6-mm fixture diameter improves stress distribution and reduces cortical stress by up to 27.5%. Axial loading is more favorable for implant longevity than non-axial loading.
Sabri et al. 2020 [70]	ND	Supra structure, fixture, and maxilla jaw (cortical bone and cancellous bone)	Fixture body shape, thread depth, and bone classification	Von Mises stress	Full osseointegration at the implant-bone interface. Maxillary cortical bone at the outer oral cavity and nasopharynx surfaces is fixed in all directions.	Tapered fixture produce higher von mises stress in cortical bone than cylindrical fixture. A 0.35-mm thread depth yields the lowest stress, while a 0.45-mm depth (tapered) produces the highest stress in type iii-iv bone.
Lee et al. 2021 [71]	Statistical Tests: Shapiro-Wilk Test, Kruskal-Wallis One-Way Test, Mann-Whitney U Test, Bonferroni Correction	Crown, cement layer, abutment screw, abutment, fixture, cortical bone, cancellous bone, nerve canal, and cylindrical part surrounding implant	Fixture diameter, fixture level, cancellous bone density, and loading	Von Mises stress, microgap, fatigue failure	Full osseointegration at the implant-bone interface. Abutment-screw interface has a friction coefficient (µ = 0.441), abutment-fixture (µ = 0.16), fixture-cement (µ = 0.25). Mesial and distal surfaces of cortical and cancellous bone are fixed in x, y, z axes.	Tissue-level (it) connections exhibit smaller microgaps and better fatigue resistance than bone-level (ib) connections, which are more vulnerable, particularly at 3.5-mm diameters. Bone density does not significantly affect microgaps but enhances fatigue life in ib fixture.
Sumra et al. 2021 [72]	ND	Crown, crown framework, cement layer, abutment, screw abutment, fixture, cortical bone, and cancellous bone	Fixture thread shape, fixture diameter, and loading	Von Mises stress, strain, micromovement	All material interfaces are bonded with distinct material properties.	In d4 bone, a 6-mm diameter fixture with square threads produces the lowest stress, strain, and micromovement compared to a 4-mm diameter and v-shaped threads. Ultra-short 5-mm fixture with wide diameters are a viable alternative for atrophic ridges.
Oliveira et al. 2020 [73]	ND	Prosthesis, abutment, fixture, cortical bone, and cancellous bone	Fixture design, cortical bone thickness, and cancellous bone density	Von Mises stress, strain distribution	Full osseointegration at the implant-bone interface. Outermost alveolar bone is fixed.	Vega + implants exhibit more homogeneous strain distribution and the lowest cortical stress, while essential cone implants show higher stress. Platform-switching in vega and vega + implants reduces cortical stress.
Tomar et al. 2020 [74]	ND	Abutment-fixture, cortical bone, and cancellous bone	Fixture thread shape and platform switching model	Von Mises stress, total displacement	Full osseointegration at the implant-bone interface without movement.	Single-threaded fixture with platform-switching produce the lowest stress (15.4 mpa) in d4 bone, optimal for the posterior maxilla.
Kalay et al. 2021 [75]	ND	Abutment-fixture, cortical bone, and cancellous bone	Fixture thread shape and loading	Von Mises stress, stress transfer parameter (stp)	Outer cortical and trabecular bone layers are fixed. Implants move only along the z-axis. Implant-bone interface has a friction coefficient (µ = 0.5).	Rectangular thread profiles yield the lowest stress distribution in fixture, while buttressed threads optimally transfer stress to bone, supporting long-term regeneration and stability.
Patwari et al. 2024 [76]	ND	Abutment, fixture, cortical bone, and cancellous bone	Fixture thread shape and loading	Von Mises stress	Distal bone is fixed in all directions. Bone base is not fully supported for bending.	Thread designs with maximum cortical stress typically yield minimum cancellous stress, and vice versa. Buttress threads produce the highest cortical stress under axial loading, square threads under 45° oblique loading. Reverse buttress threads yield the lowest cortical stress but the highest cancellous stress. V-threads produce the highest stress in the fixture body.
Park et al. 2022 [77]	ND	Crown, cement layer, abutment screw, abutment, fixture, cortical bone, and cancellous bone	Fixture diameter, fixture length, fixture level, bone classification, and loading	Distribution of apparent density, principal strains, and volume fraction values in fatigue failure region	Mesial and distal bone planes are fixed in all directions. Implant-bone, bone-bone, crown-cement, cement-abutment, abutment-screw, and abutment-implant interfaces are bonded.	Peri-implant bone stability is more influenced by initial bone conditions than fixture design. Short or narrow-diameter fixture increase excessive strain (> 3000 µε) and fatigue failure risk. Tl fixture outperform bl fixture in d4 bone.
Djebbar et al. 2022 [78]	ND	Abutment, fixture, quarter of mandible jaw (cortical and cancellous bone)	Fixture thread shape and projectile shape	Von Mises stress	Full osseointegration at the implant-bone interface. Cortical bone is embedded without displacement or rotation. Impactor moves along the z-axis with surface-to-surface interaction (µ = 0.28).	Maximum pressure occurs at the impactor-bone contact zone, with conical projectiles causing higher pressure than hemispherical ones. Triangular and trapezoidal threads produce higher pressure, while round and square threads yield lower pressure.
Rahmanivahid et al. 2020 [79]	ND	Fixture, quarter left of mandible jaw (cortical bone and cancellous bone)	Fixture design and loading	Von Mises stress, principal stress, induced strain	Full osseointegration at the implant-bone and cortical-cancellous bone interfaces. Cortical-implant and cancellous-implant interfaces have a friction coefficient (µ = 0.35). Outermost mesial and distal locations are fixed in all directions. Thermal properties are neglected.	V-shaped threads provide more even stress distribution than variable-fin designs (v-5), which increase micromovement and bone fracture risk.
Dere et al. 2021 [80]	ND	Fixture, cortical bone and cancellous bone	Fixture body shape, fixture insertion depth, measure the distance from the fixture	Von Mises stress, maximum principal stress, and minimum principal stress	Peripheral model points are fixed to prevent rigid body motion. Implants are rigid with rotational and translational movement along the z-axis. Implant insertion is modeled incrementally with constant torque.	Tapered fixture produce higher stress than cylindrical fixture, particularly in cortical bone near the fixture neck.
Didier et al. 2020 [81]	DOE: Full Factorial Design, Multi-Objective Optimization: Genetic Algorithm (NSGA-II Type), Mathematical Method: Taylor-Maclaurin Series	Abutment, fixture, cortical bone, and cancellous bone	Fixture diameter, fixture length, fixture body shape, fixture angulation, young’s modulus of the fixture, cortical bone thickness, and cancellous bone young’s modulus	Von Mises stress, stress jump at the implant-bone interface	All model component interactions are bonded. Cancellous-cortical bone is embedded. Loading is parallel to the implant axis without inclination.	Cortical thickness, fixture diameter, and fixture elastic modulus most significantly affect peri-implant stress distribution. Thin cortical bone increases stress, while larger diameters reduce stress. Lower elastic modulus minimizes stress shielding. Optimal configuration for type iv bone: taper 0.35°–2.55°, diameter 3.79–3.97 mm, length 14.96–14.99 mm, inclination 0.07°–1.25°, and modulus 60.34–62.57 gpa.
Shaukat et al. 2024 [82]	In-Vitro: Strain Gauge Analysis (SGA), Statistical Tests for SGA (not FEA): Mixed ANOVA and Post-Hoc Tukey’s Test	Crown, crown framework, screw crown/cement layer, abutment, fixture, cortical bone, and cancellous bone	Fixture thread shape and retention mode of the crown	Von Mises stress, equivalent elastic strain, and total deformation	Full osseointegration at the implant-cortical bone, implant-cancellous bone, and cancellous-cortical bone interfaces. Inferior bone boundary is fixed.	Buttress thread (bt) designs yield low strain, while trapezoidal threads (tt) produce high strain in peri-implant bone. Combining tt with cement retention and bt with screw retention increases peri-implant strain.
Moreno et al. 2023 [83]	ND	Crown, cement layer, abutment, fixture, cortical bone, cancellous bone, and mandibular canal	Fixture diameter and loading	Von Mises stress, minimum principal stress, and maximum shear stress	Implant-abutment interface prevents separation. Crown-abutment and implant-bone interfaces are bonded. Two lateral bone segments are fixed.	Extra-short 6-mm diameter fixture reduce fixture stress by ~ 38%, but increase abutment and cortical bone stress by > 200% under oblique loading, risking accelerated marginal bone resorption.
Robau-Porrua et al. 2020 [84]	Statistical Test: ANOVA; DOE: Multi-Level Factorial Statistical Design	Abutment-fixture, cortical bone, and cancellous bone	Fixture diameter, fixture length, young’s modulus of the fixture, and loading	Von Mises stress, maximum principal stress, minimum principal stress, and von Mises strain	All three degrees of freedom of cortical and trabecular bone are fixed. Bone interactions are bonded. Implant-bone interface has a friction coefficient (µ = 0.3).	Fixture diameter and length significantly affect stress and strain, including their interaction and elastic modulus.
Ishak et al. 2024 [85]	ND	Prosthesis, crown framework, abutment, abutment screw, fixture, cortical bone, and cancellous bone	Fixture length and bone classification	Maximum principal stress, von Mises stress, maximum principal strain, and total deformation	Full osseointegration at the implant-bone and cortical-cancellous bone interfaces. Implant-prosthetic component interface has a friction coefficient (µ = 0.3). Inferior cortical bone plane is fixed in all directions. Initial temperature is 27°c.	Longer fixture reduce stress in type ii bone but increase stress in type iii bone. Fixture body stress increases with fixture length. Longer fixture reduce strain and deformation at the implant-abutment interface.
Sharma et al. 2020 [86]	ND	Abutment, fixture, cortical bone, and cancellous bone	Fixture body shape, fixture thread shape, and loading	Von Mises stress	Not specified.	Tapered reverse buttress and cylindrical buttress threads produce the highest stress, while tapered reverse buttress and v-threads yield the lowest cortical stress concentration. Tapered designs with specific threads aid bone preservation through stress redistribution.

*ND* Not Described, *DOE* Design of Experiment, *RSM* Response Surface Method

*data is assumed/defined based on existing information or images

### Bone characteristics

This subsection analyzes the bone characteristics modeled in the studies to understand the biomechanical simulation context. Most studies (79.49%, 31/39) modeled the mandible, indicating that the mandible was analyzed more frequently than the maxilla (Table 4). The most frequent implant placement site was the mandibular first molar (38.46%, 15/39), followed by the mandibular molar (10.26%, 4/39) and maxillary first molar (10.26%, 4/39), indicates a significant frequency of edentulism in the posterior region. Most bone models incorporated cortical and cancellous layers (97.44%, 38/39), closely resembling physiological structures and enabling more accurate FEA simulations, while one study used cortical layers only (2.56%). However, 56.41% (22/39) of studies did not use CT scans, likely due to simplified modeling assumptions. Using non-CT models may reduce simulation accuracy in representing clinical bone anatomy. This variability could affect stress estimation, especially when combined with static loading. Figure 2 depicts the bone characterization across all studies.

### Fixture characteristics

Next, fixture characteristics were analyzed based on material, commercial product-based models, brand, abutment–fixture connection, fixture level, body shape, and thread shape, all influencing stress and strain distribution at the bone-implant interface (Table 5). Titanium dominated as the material (51.16%, 22/39), followed by Ti-6Al-4 V alloy (20.39%, 9/39) because of its biocompatibility and mechanical strength. Commercial product-based models were relatively common (56.41%, 22/39), with Straumann (39.53%, 6/39) and Nobel Biocare (11.63%, 5/39) as the frequently used industry standards. Two-piece* abutment–fiture (75%, 31/39) were more prevalent than one-piece (15%, 6/39). Bone-level (BL)* implants (44.44%, 17/39) were more common than tissue-level (TL)* (20%, 9/39), supporting posterior restorations. Tapered* body shapes (20.41%, 10/39) were slightly more used than cylindrical* (18.37%, 9/39). V-shaped threads (23.53%, 16/39) dominated, followed by square (11.76%, 8/39) and buttress threads (10.29%, 7/39). Figure 3 illustrates fixture characteristics from all studies.

### Fixture dimensional

This section evaluates reported fixture dimensions for consistency in reporting (Table 6). All studies reported fixture diameter and length (100%, 39/39). Diameters ranged from 3.0 to 6.0 mm, with 4.0 mm being the most frequent value, and lengths varied between 4.0 and 16.0 mm, with 10.0 mm as the dominant value, which is considered suitable for posterior bone height. However, thread dimension reporting, such as thread depth, width, and pitch, was less consistent. Thread width (0.1–0.8 mm, generally 0.20 mm) was only present in 12.82% (5/39) of the studies, thread depth (0.1–0.6 mm, generally 0.35 mm) in 38.46% (15/39), and thread pitch (0.4–2.4 mm, generally 0.80 mm) in 53.85% (21/39). The low reporting rate of thread dimensions reflects a research focus on diameter and length because these are easier to measure. This inconsistency affects the ability to replicate the study entirely. Figure 4 shows the range of dimensions and other findings related to the dimensions.

### Loading characteristics

The loading characteristics were explicitly analyzed to determine the simulation trend of the masticatory force on the posterior teeth, which is very important for evaluating the stress and strain distribution (Table 7). Static loading predominated (72.73%, 34/39 studies), followed by dynamic loading (9.09%, 4/39) and immediate loading protocols (6.82%, 3/39). Axial loads (50–300 N, most often 100 N) were present in 66.67% (26/39) of studies, lateral loads (17.1–100 N, most often 50 N) in 15.38% (6/39), and oblique loads (32.4–300 N, most often 100 N; angle 3°−75°, most often 45°) in 61.54% (24/39). Figure 5 summarizes the loading characteristics.

### FEA characteristics

FEA characteristics are important in assessing the accuracy of stress and strain simulations that match the research objectives (Table 8). Three-dimensional models dominated (94.87%, 37/39), which allowed biomechanical analyses closer to clinical conditions, compared to two-dimensional models (2.56%, 1/39) or unspecified (2.56%, 1/39). Isotropic, homogeneous, and linear elastic assumptions were used in 74.36% (29/39) of studies, simplifying computations but potentially overlooking bone anisotropy, possibly influencing results. Tetrahedral elements (69.57%, 29/39) were more common in the element shape descriptions in this dental implant FEA study than hexahedral elements (4.35%, 2/39). For convergence tests, only 41.03% (16 out of 39) of the studies reported their application. Given the importance of convergence tests in FEA-based research, this is a reason for concern about numerical validity and a significant methodological shortfall. Among the 39 studies analyzed, ANSYS emerged as the most frequently employed FEA software (51.28%), followed by Abaqus (20.51%), other software represented 7.69% and COMSOL was reported in a single study (2.56%). Furthermore, 17.95% of studies did not disclose the FEA software used, indicating variability in methodological reporting across publications. These findings highlight ANSYS and Abaqus as key platforms in dental implant biomechanics research, reflecting their robustness and widespread acceptance in finite element modeling. Figure 6 summarizes the FEA characteristics, such as type of FEA, element shape, model characteristics, and convergence test reporting.

### Study details and findings

This section analyzes the focus variables in the studies. The most frequently investigated independent variables were loading (17.36%), fixture diameter (14.88%), and fixture length (10.74%) (Table 9). The most common dependent variable was von Mises stress (43.9%), followed by principal stress (15.85%), which reflects a primary focus on implant stability. Additional methods, such as statistical testing (16%) and Design of Experiments (DOE) (8%), were used to improve the quality of the research. Figure 7 summarizes the independent and dependent variables and the additional methods used in the included studies.

### Quality assessment

The researchers assessed the methodological quality of 39 studies using ROBFEAD, a specialized tool for evaluating the risk of bias in dentistry’s FEA studies. Figure 8 illustrates the distribution of methodological weaknesses per question, highlighting the lack of mechanical validation and dynamic loading: 36 of 39 studies (92.3%) did not perform mechanical validation, 35 studies (89.7%) assumed isotropic material properties, 22 studies (56.4%) did not use CT imaging, and only five studies (12.8%) applied dynamic loading.

**Fig. 9:**
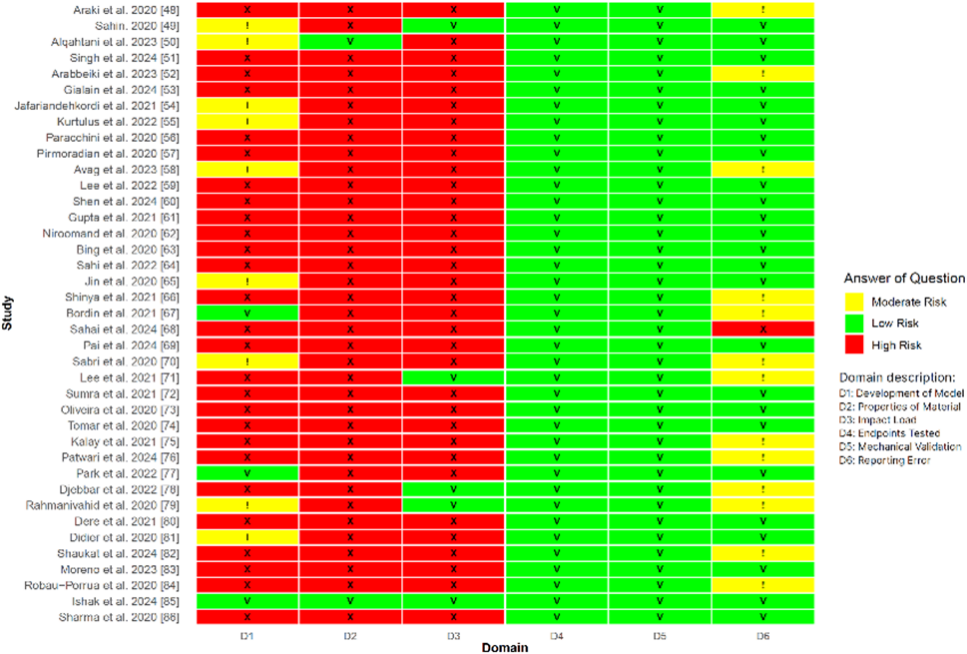
Domain-wise risk of bias assessment

The risk of bias across domains, visualized in Fig. 9, indicates that material properties (36 of 39 studies; 92.3%) and impact load (34 of 39 studies; 87.2%) exhibited the highest risk. These weaknesses reflect the oversimplification of material properties and loading conditions in FEA for implant fixture design. In the overall review, all studies showed a high risk of bias due to the large number of studies in D1 and D2 showed a high risk of bias. A high risk of bias undermines the reliability of evidence regarding the influence of fixture design on stress and strain distribution in the posterior region, necessitating caution in interpreting FEA findings. In addition, variability in study quality, such as limited application of CT imaging, dynamic loading, or others, may lead to incorrect findings for implant fixture design, undermining the credibility of FEA simulations.

## Discussion

The design of dental implant fixtures plays a critical role in determining the biomechanical stability and the distribution of stress and strain on cortical and cancellous bone in the posterior region. Parameters such as fixture level, fixture body shape, fixture thread shape, fixture diameter, fixture length, thread depth, thread width, thread pitch, thread angle, and neck model interact in a complex manner to affect implant performance. Utilizing FEA facilitates the acquisition of valuable insights into optimizing fixture designs, thereby ensuring clinical success in a range of bone conditions.

Fixture Level affects stress distribution at the implant-bone interface, with significant implications for long-term stability. Tissue-level (TL) fixture show a more even distribution of stress on the cortical bone compared to bone-level (BL) fixture [48]. BL fixture generate a maximum von Mises stress of up to 342.77 MPa under 50 N oblique loading, with the maximum tensile stress on cortical bone reaching 114.1 MPa, which approaches the cortical bone strength limit, thus increasing the risk of long-term bone resorption, although not causing failure [58]. TL fixture result in a better stress distribution at the bone-implant interface, with lower compressive stress (−43.73 MPa) on cortical bone, supported by the transmucosal part that facilitates stress redistribution [58, 59]. TL also has smaller micro-gaps at the abutment-implant interface and better fatigue resistance than BL, which is prone to fatigue failure (~ 1,000 cycles for a 3.5 mm diameter) [71]. In the bone-level variant, BLT (Straumann) results in lower stress than BLX (Straumann), but BL shows a larger volume fraction in the fatigue failure range in D4 bone compared to TL [55, 77]. Thus, in general, TL performs better than BL. However, it is crucial to consider the bone state and investigate how fixture level interacts with other fixture parameters.

Another aspect analyzed in depth is fixture body shape, which can modulate the biomechanical response. Tapered fixtures produce higher stress concentrations on cortical bone, particularly in the cervical region, than cylindrical fixtures [70, 80]. A stepped design with a length of 10 mm and a diameter of 3.75 mm is more effective in distributing stress and strain on low-density bone. It is recommended for immediate loading protocols in patients with D4 bone [51]. Tapered designs with an inverted conical neck and “nest shape” threads shift stress from cortical bone to cancellous bone, enhancing biomechanical performance in high-density bone [56]. Tapered fixtures with reverse buttress threads recorded the lowest von Mises stress on the cortical bone (2.44 MPa) under axial loading of 100 N, and tapered fixtures with V-thread under buccolingual loading of 50 N (3.28 MPa), whereas cylindrical designs with buttress threads generated higher stress (75.51 MPa) under buccolingual loading [86]. The heterogeneity of tapered design results is likely due to variations in loading conditions. Thin cortical bone significantly increases stress, whereas low elastic modulus (60.34–62.57 GPa) minimizes stress shielding and stress jump, with an optimal configuration including a taper angle of 0.35°–2.55°, a diameter of 3.79–3.97 mm, and a length of 14.96–14.99 mm for type IV bone [81]. Despite the heterogeneity, tapered fixture generally show stress and strain distribution advantages, with the optimal taper angle range of 0.35°–2.55° for type IV bone.

Further related to the fixture thread shape significantly affects stress distribution and biomechanical stability, with variations depending on the bone type and loading conditions. Square threads tend to perform better on bone compared to other thread shapes, such as V-shape, in reducing von Mises stress, strain, and micromovement, with the lowest values reaching 89.872 MPa under vertical load and 202.725 MPa under oblique load for a 6 mm diameter fixture in D4 bone [50, 68, 72]. This thread shape distributes stress evenly across the fixture surface and the cancellous bone for axial, non-axial, buccolingual, and non-axial mesiodistal loads [68]. Conversely, the reverse buttress thread excels at reducing stress in the cortical bone under oblique and axial loads but results in the highest stress in the cancellous bone and the fixture [76, 86]. Research by Sahi et al. [64] demonstrated that the MIS SEVEN implant with a reverse buttress thread had lower von Mises stress compared to the OSSTEM TS III implant with a V-shape thread in D2 and D3 bone, where high stress was found at the cervical region of cortical bone. The buttress thread minimizes stress transfer to the cancellous bone, supporting bone regeneration and long-term stability through optimal stress-shielding effects [75]. Shaukat et al. [82] found that the buttress (BT) thread produced higher strain in peri-implant bone than the trapezoidal (TT) thread, although still within the physiological range. Platform switching with a single-threaded fixture resulted in the lowest stress (15.4 MPa) in D4 cortical bone, making it the optimal design for the posterior maxilla. However, it increased micromovement (0.008524 mm without platform switching) [74]. On the other hand, V-shape threads tend to accumulate stress in the fixture body, increasing the risk of mechanical failure [76]. However, compared to a new model combining V-shape and variable fins, the V-shape performed better in distributing stress in both cortical and cancellous bone [79]. V-shape and trapezoidal threads increase pressure, whereas square and round threads generate lower pressure when subjected to impact forces on bone [78]. Arabbeiki et al.‘s [52] study on thread angle optimization revealed that the upper thread angle at the inner thread foot (66.2 ≤ α ≤ 80°) had a greater impact than the lower thread angle at the inner thread foot (β ≤ 65.4°), with the V-shape thread showing better biomechanical stability than square, buttress, or reverse buttress threads. The heterogeneity of these findings indicates that thread design should be selected based on the clinical context and patient condition, as thread design affects the contact area with bone; however, square threads are generally more optimal than other thread shapes.

The following parameter is fixture diameter, which affects the biomechanical stability of dental implants in posterior teeth by optimizing stress distribution in peri-implant bone. As the fixation diameter enlarges, von Mises stresses in the bone and fixation overall diminish, owing to the augmented surface area of the fixture, which facilitates improved stress distribution inside the bone [50, 60, 69]. For instance, narrow-diameter Ti-Zr 3.3 mm fixture produce higher von Mises stress in cortical and cancellous bone compared to standard-diameter 4.1 mm fixture, with stress reduction of 17% (maxilla) and 31% (mandible) under vertical load and 25% (maxilla) and 13% (mandible) under oblique load, indicating a higher biomechanical risk with smaller diameters [49]. In D4 bone, a 6 mm diameter fixture produces the lowest von Mises stress, strain, and micromovement (89.872 MPa axial loading; 202.725 MPa oblique loading) compared to a 4 mm diameter fixture, making a combination of ultra-short (5 mm) fixture with wide diameters suitable for atrophic ridges or cases with limited bone preparation [72]. A 4.77 mm diameter fixture with a 12.72 mm length and 0.72 mm thread pitch was effective in optimizing a more uniform pressure distribution [62], while a 4.3 mm diameter fixture was optimal for vertical bone loss due to peri-implantitis, as bone pressure shifts to cancellous bone when vertical bone loss exceeds 10% of fixture length [61]. Increasing the diameter to 6.0 mm reduced stress at the peri-implant by approximately 50% compared to a 3.8 mm diameter at the fixture neck [66], and a 4.8 mm diameter with a wide-neck design reduced the risk of peri-implant bone resorption by 35% and strain energy density (SED) by 5–10%, making large-diameter short fixture more recommended for atrophic mandibles [53]. Conversely, narrow-diameter fixture increase the risk of fracture due to higher stress on the fixture body and abutment screw, especially with bone resorption ≥ 2 mm [63], with 3.5 mm BL fixture showing the shortest fatigue life (~ 1,000 cycles) [71]. However, ultra-short fixture with wide diameters (6 mm) can increase cortical stress (σmin + 66.3% for axial load; + σmin 125.7% for oblique load; τmax + 99.8% for axial load; τmax + 201.7% for oblique load), potentially accelerating marginal bone resorption, although reducing cancellous stress by up to 73.2% (σmin) and 71.9% (τmax) under oblique load [83]. Fixture diameter also shows higher sensitivity to maximum equivalent strain difference compared to fixture length, with an optimal diameter of 4 mm and a 10 mm length [54]. Additionally, diameter, cortical bone thickness, and elastic modulus are the most influential variables in stress distribution in peri-implant bone, with larger diameters reducing stress in bone [81]. This statement is consistent with research by Robau-Porrua et al. [84], which found that fixture diameter and length significantly affected von Mises stress and strain values, and diameter influenced stress distribution (reducing diameter increases stress concentration). However, fixture connection had a greater impact [59]. In low-quality bone (D3/D4), narrow-diameter fixture produce excessive microstrain (>3000 µε), increasing the risk of failure due to fatigue [77]. These findings confirm that fixture diameter is crucial for implant stability and minimizing biomechanical risk. The emerging trend is that larger fixture diameters improve biomechanical response and stability. However, consideration must be given to bone condition, loading, and patient clinical condition to prevent marginal bone resorption.

In addition to diameter, another important parameter is fixture length, which affects biomechanical stability and stress distribution in peri-implant bone, with varying effects depending on fixture design and bone quality. Araki et al. [48] showed that stress on the fixture body and cortical bone decreases as fixture length increases, although the fixture-level design has a more dominant effect. For example, increasing the fixture length from 4 mm to 6 mm can reduce mechanical stimulation on cortical and trabecular bone by 18–26% [53] A 10 mm fixture generates lower micromovement, von Mises stress, and strain than a 6 mm fixture in low-density bone (D4) [51]. Additionally, in specific cases, a 13 mm length combined with sinus grafting in the posterior maxilla reduced shear stress by 35.75% in cortical bone and 51% in trabecular bone compared to a 7 mm fixture, making this combination with cemented crown retention recommended for posterior maxilla rehabilitation [67]. Increasing the length from 10 mm to 11 mm with a 4 mm diameter reduced the maximum equivalent strain difference by 50.2% (from 44.2 µε to 22.2 µε) [54]. An 11.5 mm length with a 0.8 mm thread pitch showed the lowest sensitivity and optimal stress values for cancellous and cortical bone [57]. Niroomand et al. [62] reported that a 12.72 mm length with a 4.77 mm diameter and 0.79 mm thread pitch significantly provided a more uniform stress distribution, reducing the likelihood of bone resorption. Increasing length from 9 mm to 16 mm also reduced peri-implant stress by 16–22%, although the effect was less significant than diameter [66]. Didier et al. [81] suggested that an optimal configuration of 14.96–14.99 mm length, with an inclination angle of 0.07°–1.25° and an elastic modulus of 60.34–62.57 GPa, increased load transfer and reduced stress on type IV bone. However, in type III bone, Ishak et al. [85] found that longer fixture increased stress in peri-implant bone, although reducing strain and total deformation in the implant-abutment system. In type II bone, longer fixture reduce stress. Short fixture or narrow-diameter fixture generate excessive strain (>3000 µε), increasing the risk of fatigue failure [77]. The interaction between fixture length and diameter significantly affects von Mises equivalent stress and von Mises strain [84]. Therefore, longer fixture generally improve biomechanical stability but must be adapted to bone quality and other fixture design factors to minimize the risk of bone resorption and biomechanical failure.

Focusing on thread characteristics, thread depth, width, and pitch specifically and measurably influence stress distribution. Thread depth in the range 0.25 < TD < 0.34 mm and thread width (0.25 ≤ TW ≤ 0.28 mm) has a more significant impact than pitch (0.8 ≤ TP ≤ 0.88 mm) in stimulating peri-implant bone [52]. A thread depth of 0.35 mm produces the lowest stress, while 0.45 mm in tapered fixture increases stress in cortical bone in type III and IV bones [70]. A pitch of 0.7 mm with a 13 mm length records the lowest stress on the implant-abutment, cortical, and cancellous bone. In comparison, optimal fixture length and thread pitch with the lowest sensitivity are 11.5 mm and 0.8 mm, with fixture length having greater sensitivity than pitch in cancellous and cortical bones [57]. Furthermore, thread width (0.18 mm) and thread crest angle (33.48°) help reduce maximum total deformation in the fixture, while thread depth (0.27 mm) contributes to reducing von Mises stress in cancellous bone [62]. The optimal combination of pitch is 0.79 mm with a 12.72 mm fixture length and a 4.77 mm diameter, as it provides a more uniform distribution, enhances stability, and reduces bone resorption [62]. Although these parameters consistently respond to biomechanical stimuli, variations still require thread design adjustments based on the clinical context.

Switching to neck design, neck models affect stress distribution in marginal bone, with implications for bone resorption. The microthread (T-neck) design effectively reduces shear stress and marginal bone resorption in healthy bone, with a stress penetration depth of 0.6 mm compared to 0.8 mm in S-neck designs [65]. However, the effectiveness of microthreads decreases as bone resorption increases (0.75 mm), with S-neck designs showing more uniform compression stress distribution at the bone apex [65]. Since these results depend on the bone resorption level, the neck model should be adapted to the patient’s clinical condition to ensure long-term stability.

The quality assessment of the reviewed studies through the ROBFEAD framework reveals methodological limitations that affect the reliability of FEA simulations in predicting dental implant performance. The lack of experimental validation for the FEA model may lead to unrealistic predictions of implant stability. The assumption of isotropic bone material can potentially distort stress and strain distribution. The representation of static loading limits the model’s ability to capture biomechanical responses over long-term masticatory cycles, which is critical for assessing fatigue resistance. The simulation’s lack of ability to represent individual anatomical variations due to the lack of patient-specific imaging-based modeling and mesh convergence testing decreases numerical accuracy, especially in cases of atrophy or low-density bone (D4). This review synthesizes comparative mechanical effects of fixture geometry under specified boundary conditions. Because in-silico models often employ simplified material laws [55, 59], static or simplified loading, and may lack mechanical or clinical validation [57], their estimates should be interpreted as hypothesis-generating rather than clinically directive. We mitigated this by applying ROBFEAD, a validated risk-of-bias tool for dental FEA [47], and avoiding claims beyond stress/strain trends. Nevertheless, external validity is limited: implant survival, marginal bone level changes, and biological complications depend on additional clinical determinants (site anatomy, surgical protocol, prosthesis design, hygiene, parafunction) [53, 59] that FEA does not capture. Therefore, the results require careful interpretation due to the risk of biases. This confidence derives from strict adherence to PRISMA 2020 and SWiM guidance, combined with structured ROBFEAD appraisal.

Clinically, FEA results should be integrated with longitudinal clinical data and in vitro testing to verify stress distribution and minimize the risk of implant failure due to discrepancies between simulation and biological reality. Where available, clinical and bench evidence offer context for our findings. Randomized and comparative data indicate that short (≤ 6 mm) implants can achieve 5-year survival comparable to ≥ 10 mm implants in native bone, although results vary by indication and protocol [1]. Similarly, posterior-region meta-analyses show no consistent survival difference between narrow and regular diameters and heterogeneous effects on marginal bone loss [2, 3]. These outcomes cannot be inferred from FEA alone and require triangulation with dynamic fatigue tests, such as ISO 14,801 [4] and well-designed clinical studies. Future reviews should therefore integrate three evidence streams, such as FEA, standardized mechanical testing, and clinical outcomes reported transparently per SWiM [45].

Although all studies reported fixture diameter and length, thread geometry parameters, particularly depth and width were frequently omitted. Only 38.46% of the studies reported thread depth, and 12.82% reported thread width. This omission may introduce internal bias, as these parameters directly influence stress concentration at the bone-implant interface. Prior evidence has shown that thread depth and width significantly affect cortical and trabecular stress distribution, especially in low-density posterior bones such as D3–D4 [52, 87]. Therefore, future FEA-based implant studies should implement standardized reporting of thread design, including depth, width, pitch, and angle, to improve biomechanical assessments and clinical translatability [88–90].

Future research should prioritize experimental validation of FEA models through mechanical in vitro testing [91] or correlation with prospective clinical data [92] to ensure accurate biomechanical performance predictions. Patient-specific bone modeling, such as CT imaging [93], and the characterization of anisotropic materials [94] will improve biological fidelity [95]. In addition, dynamic loading simulations that mimic the physiological mastication cycle [96] are essential for evaluating the long-term efficacy of implants and helping clinicians select implant configurations tailored to each patient’s clinical needs, especially in complicated cases such as posterior maxillary rehabilitation [97].

## Conclusion

This systematic study aimed to assess how dental implant fixture design affected the posterior region’s biomechanical stability and stress distribution, a critical area due to the high prevalence of tooth loss, using FEA. Narrative synthesis was performed by grouping studies based on fixture design parameters (fixture level, body shape, thread characteristics, diameter, length, and neck model) to assess their impact on biomechanical stability. Tissue-level fixtures generally provide better stress distribution than bone-level fixtures, particularly in reducing cortical bone stress and improving fatigue resistance. The tapered body shape showed an advantage in low-density bone by redistributing stress to less dense bone, although the optimal taper angle varied according to bone condition. Square threads consistently outperformed other thread shapes, such as V-shape or inverted buttress, in minimizing stress and micro-motion, particularly in D4 bone. Thread depth, width, pitch, and angle further modulate biomechanical outcomes, with specific configurations optimizing stability. Larger fixture diameters and longer lengths improve stability by reducing peri-implant stress, although excessive dimensions may increase cortical stress under certain conditions. The micro-threaded neck design effectively reduces marginal bone resorption in healthy bone but requires adaptation to bone resorption rates. The narrative synthesis, guided by SWiM, did not use quantitative statistical analysis, limiting the generalizability of the findings and requiring further clinical confirmation. The reviewed studies exhibited significant limitations, including a high risk of bias identified through the ROBFEAD framework, mainly due to the lack of mechanical validation, assumption of isotropic materials, and limited use of dynamic loading or patient-specific imaging. These methodological shortcomings require careful interpretation of the FEA results. To reduce bias, future studies should standardize reporting of thread dimensions, apply CT-based modeling for patient-specific bone anatomy, and perform mesh convergence testing. Further research should characterize anisotropic material properties to reflect biological complexity and simulate dynamic loading to mimic the physiological masticatory cycle to guide the development of customized implant designs for complex posterior restorations. Future FEA studies should standardize the reporting of thread design parameters, particularly thread depth, and width, which are critical to accurate stress analysis and implant stability, especially in low-density posterior bone. Inconsistent reporting of these variables may lead to internal bias and limit clinical applicability. It is highly recommended to establish a minimum reporting framework for thread geometry.

### Statement of originality

The authors declare that this manuscript is original, has not been published before and is not currently being considered for publication elsewhere. The authors confirm that the manuscript has been read and approved by all named authors and that there are no other persons who satisfied the criteria for authorship but are not listed. The authors further confirm that the order of authors listed in the manuscript has been approved by all of us. The authors understand that the Corresponding Author is the sole contact for the Editorial process. The corresponding author is responsible for communicating with the other authors about progress, submissions of revisions and final approval of proofs.

## Data Availability

All data generated or analyzed during this study are included in this published article. No additional datasets were generated or analyzed beyond the contents of the manuscript.

## Abbreviations

BL: Bone-level
CT: Computed tomography
FEA: Finite element analysis
PICO: Population, intervention, comparison, and outcome
PRISMA: Preferred reporting items for systematic reviews and meta-analyses
ROBEFEAD: Risk of bias in studies using finite element analysis in dentistry
SWiM: Synthesis Without Meta-analysis
TL: Tissue-level
